# Phosphate removal by spent low temperature shift catalyst through process optimization and mechanistic study

**DOI:** 10.1038/s41598-026-64790-4

**Published:** 2026-09-03

**Authors:** Fatma E. A. Hashem, Abeer M. Shoaib, Walaa A. E. Omar, Mai H. Roushdy

**Affiliations:** 1 Chemical Engineering Department, Faculty of Engineering, The British University in Egypt, El-Shorouk City, Cairo, 11837 Egypt; 2 Petroleum Refining and Petrochemical Engineering Department, Faculty of Petroleum and Mining Engineering, Suez University, Suez, 43512 Egypt

**Keywords:** Treatment of waste, Value calculation, Process improvements, Circular economy, Low-temperature shift spent catalyst, Removal of phosphate ions, Chemistry, Engineering, Environmental sciences, Materials science

## Abstract

Natural water bodies become eutrophic due to phosphate pollution from industrial and agricultural sectors. This research addresses waste disposal and water pollution at the same time by recycling spent low-temperature shift catalyst (LTSSC) from the fertilizer industry as a sustainable phosphate adsorbent. High surface area (247 ± 5 m^2^/g), mesoporous structure, and many metal oxide sites (CuO 49%, ZnO 32.2%, and Al_2_O₃ 13.9%) were shown by characterization using XRF, XRD, BET, SEM–EDX, FTIR, particle size analysis, and zeta potential. Under optimum conditions (contact time 1.64 h, dose 5 g/L, initial concentration 9.58 mg/L, stirring 278 rpm), Response Surface Methodology optimization achieved 92.95 ± 2.8% phosphate removal. Chemisorption via inner-sphere complexation is established by endothermic spontaneous thermodynamics (ΔH° = 18.42 ± 1.3 kJ/mol, ΔG° = − 7.56 ± 0.6 kJ/mol, ΔS° = 36.6 ± 2.5 J/mol·K), FTIR-detected M–O-P bonds, XRD-confirmed crystalline AlPO₄ formation, and Langmuir isotherm (qmax = 0.174 ± 0.005 mg/g). Over 15 cycles, regeneration using 0.1 M NaOH demonstrated > 75% capacity retention. Heavy metal leaching tests showed concentrations below WHO/USEPA limits. The treatment cost, according to techno-economic study, is $0.52/m^3^, which is less than that of commercial adsorbents. This study shows how to use a circular economy method to address two environmental concerns by turning hazardous industrial waste into usable phosphate adsorbent.

## Introduction

Phosphorus is a very important element for life, but it has also become one of the biggest environmental pollutants due to human activities. A major source of pollution is the discharge of excessive phosphate from agricultural runoff, domestic sewage, and industrial effluents particularly those of fertilizer factory and petrochemical industries which have caused severe damage to aquatic ecosystems all over the world^1,2^. The main effect of eutrophication is the depletion of dissolved oxygen in water which results in the growth of harmful algae, fish kills, and loss of biodiversity^3–5^.

To address eutrophication, regulatory bodies have set phosphate discharge restrictions that are very stringent: the USEPA requires 0.025–0.1 mg/L^6^, WHO set a limit at 0.1 mg/L, and Egypt's Environmental Law allow up to 10 mg/L in case of industrial effluents. These standards can be fulfilled only with highly effective tertiary treatment processes that go beyond the ordinary primary and secondary wastewater treatment.

Adsorption has emerged as the preferred tertiary treatment method for phosphate removal due to its simplicity, cost-effectiveness, and high efficiency. Numerous adsorbents have been investigated, including activated carbon, activated alumina, ion-exchange resins, granular ferric hydroxide, lanthanum-modified materials, and biochar^7–15^. However, purpose-designed commercial adsorbents carry high procurement costs ($1,200–4,500/ton), limiting widespread application particularly in developing countries, driving research into sustainable low-cost alternatives from industrial waste streams.

Metal oxide-rich industrial wastes are an excellent raw material for the development of sustainable adsorbents. This offers a great opportunity to combine the principles of the circular economy and water treatment goals. Spent catalysts from the synthesis of chemicals are very rich in transition metal oxides and possess great surface properties. So far, spent catalysts have been used to some extent for the removal of heavy metals and dyes from the environment^16–25^, however, the use of spent low-temperature shift (LTS) catalysts for phosphate removal has not yet been thoroughly investigated.

LTS catalysts, especially those utilized in ammonia synthesis, methanol production, and hydrogen generation at 180–250 °C, mainly consist of CuO (40–55%), ZnO (25–35%), and Al₂O_3_ (10–20%)^26,27^. However, after 2–5 years these catalysts get deactivated due to sintering and poisoning processes. As a result, they become spent and are classified as hazardous industrial waste. Therefore, disposal of such waste remains a significant burden for the fertilizer manufacturers though these catalysts still contain a high percentage of metal oxides with surface activity.

There remain major knowledge gaps in using LTSSC-based phosphate adsorption. For example, the main removal mechanism is still unknown; no one has done a systematic optimization of parameters; the long-term regenerability performance over multiple cycles hasn't been done; and the techno-economic feasibility compared to commercial adsorbents hasn't been evaluated.

This study addresses these knowledge gaps through: (1) complete physicochemical characterization (XRF, XRD, BET, SEM–EDX, FTIR, particle size, zeta potential); (2) systematic optimization via Response Surface Methodology with Box-Behnken Design; (3) mechanistic elucidation through isotherm, kinetic, and thermodynamic modeling with spectroscopic evidence; (4) extended 15-cycle regeneration assessment; (5) environmental safety evaluation via metal leaching tests; and (6) preliminary techno-economic assessment. This work contributes to both phosphate removal science and circular economy strategies for hazardous industrial waste valorization.

## Materials and methods

All experimental work was performed in triplicate (n = 3). Data are presented as mean ± SD, where applicable. The model coefficients were obtained using nonlinear regression, considering a 95% confidence interval.

### Chemicals

All experiments were performed with analytical reagent-grade chemicals (≥ 99% purity) as shown in Table 1, and no purification was done. For the preparation of synthetic phosphate solutions, sodium dihydrogen phosphate monohydrate was used, whereas phosphate analysis with the molybdenum blue method was carried out using ammonium molybdate, L-ascorbic acid, and sulfuric acid. Sodium hydroxide and hydrochloric acid were utilized for pH adjustment and regeneration studies, and deionized water was used throughout.

**Table 1:** Chemical purity and suppliers.

Chemical	Purity	Supplier	Purpose
NaH₂PO₄·H₂O	≥ 99.5% (ACS)	Sigma-Aldrich	Synthetic phosphate solution preparation
NaOH	≥ 98% (pellets)	Merck	pH adjustment, regeneration
HCl	37% (AR)	Fisher Scientific	pH adjustment
Ammonium molybdate tetrahydrate	≥ 99.0%	Sigma-Aldrich	Phosphate analysis (colorimetric)
L-Ascorbic acid	≥ 99.0%	Merck	Phosphate analysis (reducing agent)
Sulfuric acid	95–98%	Fisher Scientific	Phosphate analysis (acidification)

### Catalyst preparation and activation

The catalyst type (Cu–Zn-Al low-temperature shift catalyst) is derived from an industrial source (ammonia production plant) after operating for a typical life (3–5 years) due to deactivation mechanisms (carbon deposition, sintering, sulfur poisoning). Low-temperature shift spent catalyst (LTSSC) was taken from Abu Zaabal Fertilizers Company (Qalyubia Governorate, Egypt). To eliminate impurities, the catalyst was washed by 3 cycles with deionized water (1:10 solid: liquid ratio), vacuum filtration, and washing until pH neutral. The catalyst was dried at 110 °C for 12 h in an air oven with mass loss monitoring. The grinding of the catalyst was done using Ball mill conditions (300 rpm, 2 h, 10 mm zirconia balls, ball-to-powder ratio 10:1). The ground catalyst was sieved so the particle size fraction selection (< 75 μm retained). The selected fraction was heated for thermal activation with the heating profile (5 °C/min ramp to 400 °C, 2 h isothermal hold) at atmosphere (air), so the mass loss during calcination became (2.97% LOI). Subsequently, the activated material was kept in a desiccator to avoid moisture uptake and carbonation, resulting in an adsorbent with better surface area and phosphate adsorption capacity.

### Adsorbent characterizations

A thorough physicochemical characterisation was carried out. The American Society for Testing and Materials' ASTM standard C114-18 was used to determine the elemental composition using X-ray fluorescence spectroscopy^28^, and crystallographic phases were studied using X-ray diffraction using an Empyrean-Malvern Analytical-Netherlands diffractometer^29^. Nitrogen adsorption and desorption tests using the BET technique were used to examine the surface area and pore structure^30^. Scanning electron microscopy and energy-dispersive X-ray spectroscopy were used to examine the surface morphology and elemental distribution^31^. Functional groups were identified by FTIR spectroscopy^29,32^, and the particle size distribution was determined by laser diffraction analysis using ASTM E 11/2009^33^ and ASTM D 422/2007^34^ standards. Electrophoretic light scattering was used to assess surface charge properties, such as zeta potential and point of zero charge, over a wide pH range. Thermogravimetric analysis was used to track changes in composition and thermal stability^35,36^.

### Batch adsorption experiments

250 mL Erlenmeyer flasks containing 100 mL of synthetic phosphate solutions prepared from a NaH₂PO₄·H₂O stock were used for batch adsorption tests. The pH of the solution was adjusted to 8.0 ± 0.1, which is the typical wastewater pH. An orbital shaker with a temperature-control capability was used to administer a precise dose of LTSSC while simultaneously and continuously swirling the mixtures at predetermined speeds. At fixed time points, samples were drawn, then filtered for adsorbent removal, followed by their immediate phosphate analysis. All experiments were done three times at 25± 1 °C, and control tests showed that phosphate loss due to factors other than the adsorbent was negligible.

Phosphate concentrations were determined through a modified molybdenum blue spectrophotometric method with increased sensitivity. Samples that were filtered were reacted with sulfuric acid, ammonium molybdate, and ascorbic acid to form a blue color, and the absorbance was recorded at 880 nm with a UV–Vis spectrophotometer. The calibration curves were made each day over an extensive concentration range, showing good linearity, and the method attained a low detection limit^37^.

The phosphate removal efficiency (%) was determined by Eq. (1):

1 $$\mathrm{R}\mathrm{e}\mathrm{m}\mathrm{o}\mathrm{v}\mathrm{a}\mathrm{l} \mathrm{\%}=\frac{{\mathrm{C}}_{0}-{\mathrm{C}}_{\mathrm{t}}}{{\mathrm{c}}_{0}}\times 100$$

where C₀ and Ct are the initial phosphate concentration and the phosphate concentration at time t (mg/L) respectively.

### Experimental design and statistical optimization

Using Design-Expert software (Version 13, Stat-Ease Inc., USA), a Response Surface Methodology based on a Box Behnken Design was carried out to methodically improve phosphate adsorption settings. Because it required fewer experimental runs, excluded factor combinations at the extremes, and was better suited for adsorption processes that might function poorly under extreme circumstances, the Box Behnken Design was chosen over the Central Composite Design^38^.

Four independent variables included were: contact time (h); adsorbent dose (g/L); initial concentration of phosphate (mg) per unit volume (L); and stirring rate (rpm). The contact time (0.5–4 h) to capture adsorption kinetics from the rapid initial uptake to equilibrium. The adsorbent dose (1–5 g/L) to achieve a good removal efficiency while keeping the cost of material low. The initial phosphate concentration (0.1–10 mg/L). The stirring rate (200–450 rpm) to allow effective mixing without causing excessive shear and high energy consumption^39–41^. The study considers environmental critical concentrations as the USEPA's recommended level of 0.1 mg/L^6^ and the World Health Organization (WHO)^7^ recommends that surface water from becoming eutrophic. The Egyptian Environmental Law No. sets a limit of 10 mg/L for discharge. 4^8^. The solution pH was controlled at 8.0 ± 0.1 to mimic the regular condition of wastewater.

Precisely weighed LTSSC was added, and the whole system was maintained stirring with the help of a temperature-controlled orbital shaker at the given speeds. Samples were regularly drawn, filtered to separate adsorbent particles, and subjected to phosphate analysis without delay.

### Adsorption isotherm

The way an adsorbate is distributed at equilibrium between the liquid and solid phases at a constant temperature is revealed by adsorption isotherms. They provide information about the adsorbent's surface characteristics, adsorbent-adsorbate interactions, and adsorption capability. We experimented with various starting phosphate concentrations (1–20 mg/L) to obtain isotherms, but we kept the adsorbent dose (3 g/L), pH (8.0), temperature (25 °C), and stirring rate (300 rpm) constant until the equilibrium was reached.

By fitting the adsorption experimental data with many isotherm models, the behavior of phosphate adsorption was investigated. To determine the maximum adsorption capacity, adsorption energy, and separation factor for assessing the favorability of adsorption, a Langmuir isotherm model—which presupposes monolayer adsorption on a uniform surface—was fitted. Multilayer adsorption on a heterogeneous surface was also described using the Freundlich model, where the constants stand for the surface heterogeneity factor and the adsorption capacity, respectively. Assuming that the heat of adsorption reduces linearly with increasing surface coverage, the Temkin model took adsorbent adsorbate interactions into account. Additionally, by evaluating the mean free adsorption energy, the Dubinin Radushkevich isotherm was employed to distinguish between possible chemical and physical adsorption, enabling a comprehensive comprehension of the nature of phosphate adsorption on the material. The earlier study mentioned and explained the models for the adsorption isotherm^42–45^.

### Adsorption kinetics study

The duration required for the system to attain equilibrium, rate-determining steps, and adsorption mechanisms can all be better understood by kinetic studies. To ascertain the rate and mechanism of phosphate adsorption, kinetic experiments were carried out under specific conditions. These conditions included an initial phosphate concentration of 10 mg/L, an adsorbent dose of 3 g/L, a solution pH of 8.0, a temperature of 25 °C, a stirring speed of 300 rpm, and sampling intervals ranging from 5 min to 6 h.

There were five kinetic models used. Adsorption regulated by the availability of vacant surface sites was modeled using the pseudo-first-order model. Chemisorption involving electron sharing or exchange between the adsorbent and phosphate species was modeled using the pseudo-second-order model. Chemisorption on heterogeneous surfaces was studied using the Elovich model, where the activation energy decreases with increasing surface coverage. The intraparticle diffusion process was examined using the Weber-Morris model to determine if the transport of adsorbate molecules to the surface happens through multiple steps or if the diffusion of adsorbate molecules inside the pores is the rate-limiting phase. The Boyd model was also used to differentiate the two mechanisms of intraparticle diffusion and film diffusion, thus giving a deeper comprehension of the major mass-transfer phenomena during phosphate adsorption. The models for adsorption kinetics were mentioned in the previous research^42–45^.

### Thermodynamic studies

Thermodynamic studies help reveal whether phosphate adsorption on the adsorbent is spontaneous or not, the heat effects during the reaction, and the entropy change of the system resulting from adsorption. These factors give an insight into the nature of the interactions between adsorbent and adsorbate. Adsorption experiments varying temperature were performed at 15, 25, 35, and 45 °C, with other operating conditions being constant, such as initial phosphate concentration of 10 mg/L, adsorbent dose of 3 g/L, solution pH of 8.0, stirring rate of 300 rpm, and a contact time of 4 h.

The changes in Gibbs free energy (ΔG°), enthalpy (ΔH°), and entropy (ΔS°) were estimated to determine the spontaneity, heat, and randomness level of the adsorption process. First, the distribution coefficient K_d_ that quantifies the distribution of phosphate between the solid and liquid phase at equilibrium was obtained from the equilibrium adsorption data, and then ΔG° was calculated using the basic thermodynamic equations. Afterward, the van't Hoff plot was used to derive ΔH° and ΔS° from the slope and intercept, respectively. The understanding of these parameters revealed if the adsorption was spontaneous, if it was endo- “positive ΔH°” or exothermic “negative ΔH°”, and if it was accompanied by an increase of the disorder at the solid–liquid interface. When ΔG° is negative, it means that the adsorption process occurs naturally. Moreover, the further the values are negative, the more spontaneous the process is. Positive ΔS° means the increased randomness of the adsorption process at the liquid–solid interface. The models for adsorption thermodynamics calculations were mentioned in the previous research^46,47^.

### Effect of pH on adsorption

The pH of the solution is a key variable that affects phosphate adsorption in two main ways: it changes the adsorbent surface charge, and it changes the forms of phosphate ions in the solution. The influence of pH on phosphate adsorption was studied over a wide range (pH 3–11) to include all the different species of phosphate. At the beginning of all experiments, phosphate concentration kept around 10 mg/L, adsorbent dosage 3 g/L, meanwhile temperature 25°C. The mixing speeds for phosphate solutions and adsorbent were 300 rpm while a 4-h time of contact was allowed to facilitate interaction. pH of the solution was measured before and after adsorption by a calibrated pH meter (Mettler Toledo SevenCompact, Switzerland).

Phosphates in variable ionic forms depending on the pH. Species distribution was determined from the Henderson-Hasselbalch equation taking dissociation constants (pKa values) into consideration. For example, pKa1 = 2.15 (corresponding to the reaction ${\mathrm{H}}_{{\mathrm{3}}} {\mathrm{PO}}_{4} \, \rightleftharpoons \,{\mathrm{H}}_{2} {\mathrm{PO}}_{4}^{ - } \, + \,{\mathrm{H}}^{ + }$), pKa_2_ = 7.20 (corresponding to the reaction ${\mathrm{H}}_{{\mathrm{2}}} {\mathrm{PO}}_{4}^{ - } \, \rightleftharpoons \,{\mathrm{HPO}}_{4}^{{2 - }} \, + \,{\mathrm{H}}^{ + }$, and pKa_3_ = 12.35 (corresponding to the reaction ${\mathrm{HPO}}_{4} ^{{2 - }} \rightleftharpoons \,{\mathrm{PO}}_{4} ^{{{\mathrm{3}} - }} \, + \,{\mathrm{H}}^{ + }$). At pH below 2.15, phosphate exists mainly as phosphoric acid, H_3_PO₄; from pH 2.15 to 7.20, the chief species is the monovalent dihydrogen phosphate ion, H₂PO₄^−^; at pH 7.20 to 12.35, the major species are phosphates of divalent hydrogen phosphate ion, HPO₄^2−^; and at pH greater than 12.35, phosphate is mostly in the form of the trivalent phosphate ion, PO₄^3−^. Knowledge pH-dependent effect on the adsorption behavior is important to understand the presence of these species^48^.

### Regeneration and reusability studies

Regeneration studies were also carried out through screening tests involving different regeneration solutions such as sodium hydroxide (NaOH) in five different concentrations (0.01, 0.05, 0.1, 0.5, and 1.0 M), hydrochloric acid (0.1 and 0.5 M), and sodium chloride (0.5 and 1.0 M). As per initial observations regarding high (> 90%) desorption yield with low structural damage, 0.1 M NaOH was selected as the preferred solution. To perform an in-depth study on the effectiveness of regeneration solutions, four effective regeneration techniques were chosen. Two alkaline techniques (0.1 M and 0.5 M NaOH), one salt technique (1 M NaCl), and one heat technique (400 °C) were selected, as listed below.

#### Alkaline regeneration—0.1 M NaOH (primary method)

Adsorbent reuse through regeneration is critical for determining its economic feasibility. A comprehensive test involving fifteen cycles was undertaken to ascertain long-term stability and determine probable modes of deactivation. Adsorption cycle studies were conducted under a starting phosphate concentration of 10 mg/L and an adsorbent dosage of 3 g/L. The pH of the solution was adjusted to 8.0. All experiments were conducted at a fixed temperature of 25 °C. Additionally, the solutions were subjected to stirring at a rate of 300 rpm. A reaction time of four hours was adopted to allow adequate interactions between phosphate ions and the adsorbent.

Post each adsorption cycle, the LTSSC containing the phosphate was filtered and washed two times using distilled water. Chemical regeneration was performed using 0.1 M sodium hydroxide (NaOH) as the regenerating agent at a solid-to-liquid ratio of 1:50 (w/v), with a contact time of 2 h at 25 °C under magnetic stirring at 300 rpm. The adsorbent was then filtered after regeneration, washed thoroughly with deionized water until the pH of the filtrate was 7–8, and dried in a vacuum oven at 105 °C for 12 h. The regenerated adsorbent was therefore used again in the next adsorption cycle under the same experimental conditions.

#### Alkaline regeneration—0.5 M NaOH (aggressive method)

A more concentrated NaOH (0.5 M, pH ~ 13.7) was decided to be used in desorption to see if extreme desorption can bring about better recovery of phosphate. All the steps were the same as those described in Sect. 2.10.1 except for the change in the NaOH concentration. In total, 5 regeneration cycles were carried out to monitor both the initial desorption efficiency and the long-term structural stability in comparison with the 0.1 M protocol.

#### Salt desorption—1 M NaCl (mild alternative)

Using 1 M NaCl, a less aggressive option, ionic strength-induced desorption was studied. This method is based on ion exchange, where chloride ions compete electrostatically and replace phosphate. Used adsorbent was mixed with 1 M NaCl (1:20 g/mL) at 25 °C and stirred at 200 rpm for 4 h (longer time due to less effective desorption force). The adsorbent was washed 5 times with deionized water and then dried at 80 °C for 12 h. Five cycles were conducted.

#### Thermal regeneration—400 °C (high-energy method)

Thermal destruction of the adsorbed phosphate was investigated by heating the saturated adsorbent in a muffle furnace at 400 °C for 2 h (heating rate 5 °C/min). This temperature breaks down phosphate complexes without the Cu–Zn-Al oxide framework getting significantly sintered (major phase transformation occurs > 500 °C based on TGA–DSC). After cooling to room temperature in a desiccator, the adsorbent was used for the next cycles. Due to very high energy consumption, only three cycles were run.

#### Comparative performance evaluation

For each method, we tracked: (1) desorption efficiency per cycle DE%, mass basis); (2) capacity retention relative to the fresh adsorbent (q_n_/q_0_ × 100); (3) structural changes by XRD, FTIR, and BET after last cycle; (4) economic costs, including chemicals, energy, and processing time.

#### Phosphate recovery from NaOH regeneration solution

As part of the improvement of the circular economy of this treatment technology, phosphate recovery from the NaOH regeneration solution can be done. Potentially, recovered phosphate can be reused as fertilizer, thus effectively closing the nutrient loop and bringing economic value to the process. Researchers have conducted and compared three different methods of phosphate recovery, i.e., acid precipitation, struvite precipitation, and evaporative crystallization. For the acid precipitation technique, a two-step method was used, the first being the regeneration solution acidified with sulfuric acid to pH 4–5 to favor precipitable phosphate species, and then calcium hydroxide was added to the mixture to form calcium phosphate precipitates that were finally collected. The struvite precipitation technique recovered phosphate as magnesium ammonium phosphate by adding magnesium chloride and ammonium chloride at an optimal Mg: NH₄:PO₄ molar ratio of 1:1:1, adjusting the pH to 9–10 with NaOH, and providing sufficient time for crystallization, thus obtaining a high-value, slow-release fertilizer. The evaporative crystallization technique was based on heating the regeneration solution up to 80 °C to concentrate phosphate to supersaturation, thus leading to the crystallization of phosphate salts, which were then gathered after the solution cooled down. Based on the previous research results in Table 2^49^.

**Table 2:** Comparative performance of phosphate recovery methods.

Method	Recovery efficiency (%)	Product purity (%)	P₂O₅ content (%)	Processing time (h)	Chemical cost ($/kg P)
Acid Precipitation	78.3 ± 3.2	82.5	18.2	2–3	0.85
Struvite Precipitation	92.6 ± 2.8	95.8	12.6	3–4	1.45
Evaporative Crystallization	68.4 ± 4.1	71.3	22.8	6–8	0.42

### Heavy metal leaching assessment

To determine the worst-case scenarios of leaching, experiments were performed in extreme conditions. Moreover, these conditions go beyond normal operational parameters so that a conservative safety assessment can be made. Studies were carried out with low pH (pH 3.0) for acidic industrial effluents, high pH (pH 11.0) for alkaline wastewater, and neutral pH (pH 7.0) for ordinary municipal wastewater. Other conservative measures included using a longer exposure time of 24 h to ascertain the maximal amount of leaching that could be achieved compared to normal leaching times, as well as exposing the sample at a higher temperature of 45 °C to simulate high-temperature operations where metals might become more soluble. After exposure, the samples were filtrated and stored in acidic condition through adding 2% HNO_3_ to ensure the stability of metals. The standard conditions used for analysis include 5 g/L adsorbent dosage, 300 rpm stirring speed, and deionized water without phosphate to eliminate any interference from phosphate ions during the metal leaching process. Metal concentration was analyzed using ICP-OES (Inductively Coupled Plasma Optical Emission Spectrometry) according to EPA Method 200.7^50^. The concentration levels of metals leached from the soil were compared against the standards set by the Egyptian Environmental Discharge Limits in Law No. 4/1994^51^ and the World Health Organization (WHO)^52^.

### Preliminary techno-economic assessment

The economic analysis that follows offers an initial feasibility study using information from laboratory-scale experiments, as well as the costs associated with standard chemical engineering equipment and operation conditions obtained from literature. These costs should be taken as rough estimates and not exact predictions for costs. Costs associated with full-scale production will vary depending on a number of location-specific conditions such as: quotations from equipment vendors (which may fluctuate by ± 30%), labor and regulatory requirements for the region, energy costs, influences of actual wastewater characteristics on the regeneration rate, economies of scale for different sized plants, and financial arrangements for capital expenditures.

The commercial viability of LTSSC in comparison to traditional adsorbents was evaluated by a thorough techno-economic analysis. The analysis's foundation and presumptions consider a medium-sized wastewater treatment plant with a daily treatment capacity of 1000 cubic meters that runs nonstop for 365 days a year, translating to an annual treated volume of 365,000 cubic meters. The treatment goal is to obtain an effluent phosphate content below 0.5 mg per liter, which necessitates a phosphate removal efficiency better than 95 percent. The influent phosphate concentration is expected to be 10 mg per liter.

#### Capital expenditure (CAPEX)

The prices for equipment and materials are entirely based on 2024 market prices in Egypt which were fetched through local suppliers but also a few international quotations (Alibaba.com for bulk equipment as an example) were taken into consideration as well. The currency conversion was done using the exchange rate of 1 USD = 30.9 EGP (average for 2024). Adsorbent supply equipment and civil works are examples of capital costs, which are one-time expenditures necessary for system installation and commissioning. In comparison to commercial adsorbents like activated alumina, which costs $1800 per ton, ion exchange resin, which costs $2500 per ton, and lanthanum modified bentonite, which costs $3200 per ton, the adsorbent material is based on low temperature spent supported catalyst obtained at zero procurement cost from a fertilizer company, which benefits from avoided waste disposal creating a waste valorization pathway. Processing costs reach $50 per ton for grinding, sieving, and thermal activation at 400 degrees Celsius. Transportation within a 50 dollars per ton, and granular ferric hydroxide costs $1500 per ton, making LTSSC roughly 21–46 times less expensive. Three adsorption tanks for continuous operation cost $150,000, pumps and piping cost $50,000, control and instrumentation cost $30,000, a regeneration system cost $40,000, installation and commissioning costs $50,000, and the total equipment cost for the adsorption-based treatment system is $320,000. With foundations and structural supports costing $80,000 and electrical connections costing $20,000, the total cost of civil work is $100,000, with a total capital investment of $420,000.

#### Operational expenditure (OPEX)

Adsorbent consumption, energy, labor, and waste disposal account for most annual operational costs, which are recurrent expenditures for system operation. Based on a dosage of 5 g per liter and a treatment capacity of 1000 cubic meters per day, the theoretical annual adsorbent demand without reuse reaches 1825 tons per year; however, reuse over 15 cycles reduces actual consumption to 121.7 tons per year, resulting in an annual LTSSC cost of 8519 dollars as opposed to 328500 dollars for activated alumina, assuming 10 reuse cycles. The annual saving on the adsorbent cost comes to 319981 dollars or 97 percent. Sodium hydroxide at a concentration of 0.1 M will be required for the regeneration process. The annual use of sodium hydroxide will be 24346 kg, and its annual cost will be 7304 dollars. The total cost of energy will come to 39065 dollars, including energy costs for mixing, 730 dollars; thermal activation of fresh adsorbent, 365100 kilowatt hours per year, which is equivalent to 36510 dollars, and pumping, 1825 dollars. Labor costs will be 60,000 dollars for four workers working two shifts, along with 10,000 dollars for maintenance, totaling 70,000 dollars per year. Disposal of spent adsorbent after fifteen cycles will amount to 121.7 tons or 12170 dollars per year. Thus, the total annual operation cost will be 137058 dollars, coming to 0.38 dollars per cubic meter of wastewater treated. Including capital cost amortized over ten years at an interest rate of five percent per year, the annualized capital cost will be 54390 dollars, making the total annual cost 191448 dollars or 0.52 dollars per cubic meter of wastewater treated.

#### Comparative cost analysis

Economic advantage of LTSSC can be clearly seen from the comparative cost analysis with commercial adsorbents, as illustrated in Table 3. Compared to conventional adsorbents, LTSSC brings about substantial savings per annum.

**Table 3:** Cost comparison (per m^3^ treated water) with commercial adsorbents.

Adsorbent	Material Cost	Regeneration	Energy	Total OPEX	Total with CAPEX	Savings vs. LTSSC	References
LTSSC	$0.023	$0.020	$0.107	$0.376	$0.524	Baseline	This work
Activated Alumina	$0.900	$0.035	$0.120	$1.185	$1.350	+ 158%	
Ion Exchange Resin	$1.370	$0.150	$0.080	$1.730	$1.950	+ 272%	^49^
Granular Ferric Hydroxide (GFH)	$0.685	$0.040	$0.110	$0.965	$1.140	+ 118%	^53^

“Commercial adsorbent prices derived from published techno-economic assessments and supplier quotes (2024 pricing)”

These preliminary economic estimates require validation through pilot-scale operation with actual wastewater to confirm regeneration frequency, reagent consumption, and equipment performance under field conditions.

### Statistical analysis

Adsorption, regeneration and leaching tests were all carried out in triplicate (= 3) to confirm reproducibility and statistical validity. The results are presented as mean ± standard deviation (SD). Where applicable, confidence intervals were determined at the 95% confidence level using Student's t-distribution. Analysis of variance (ANOVA) for optimization experiments was executed with Design Expert software (version 13) with statistical significance set at *p* < 0.05 were regarded as statistically significant. Non-linear regression was used to fit the parameters of the models (isotherm, kinetic and thermodynamic) through OriginPro software, whereby the uncertainties correspond to 95% confidence intervals from the regression analysis. The statistical significance of difference between experimental conditions was assessed by one-way ANOVA followed by Tukey's post-hoc test. Figures show that all error bars represent standard deviation of triplicate measurements.

### Software packages

The following software packages were used in this study: Design-Expert (version 13.0, Stat-Ease Inc., Minneapolis, USA; www.statease.com) to design experiments, optimize them with response surface methodology, and perform ANOVA statistical analysis; OriginPro (Version 2024b, OriginLab Corporation, Northhampton, USA; www.originlab.com) for kinetic, isotherm and thermodynamic modeling, data processing, figure preparation; HighScore Plus (Malvern PANalytical, Almelo, Netherlands; www.malvernpanalytical.com) for XRD phase identification and crystallographic analysis; Zetasizer Software (Version 8.02, Malvern PANalytical, Worcestershire, UK; www.malvernpanalytical.com), for particle sizing and zeta potential measurements; and EDAX APEX (Version 2.0, EDAX LLC/AMETEK Inc., Mahwah, USA; www.edax.com) for SEM–EDX elemental analysis and mapping.

### Generative AI disclosure

The authors of this manuscript utilized ChatGPT (OpenAI) primarily to generate the graphical abstract. The initial AI-generated graphical abstract was thoroughly checked and approved by the authors. Throughout writing the manuscript, generating data, and performing data analysis and other processes such as figure creation and statistical computation, no use was made of generative AI tools. Scientific content, results, discussion and conclusions were solely the product of the authors' work."

## Results and discussion

### Characterization of LTSSC solid waste

#### Chemical analysis (XRF analysis)

The elemental composition of LTSSC by XRF is given in Table 4. The used catalyst has very high levels of CuO (49.0%), ZnO (32.2%), and Al_2_O_3_ (13.9%), which together make up 95.1% of the total composition. These metal oxides are the main active components that were present in the fresh LTS catalyst formulation. The composition is very similar to that of fresh low-temperature shift (LTS) catalysts, which indicates that the main active phases are still almost intact even after industrial use. On the other hand, minor changes in the Cu: Zn:Al ratio with respect to fresh catalysts may be due to structural and chemical modifications attributed to long-term operation, such as sintering, phase transformation, or surface poisoning.

**Table 4:** Chemical analysis of LTSSC.

Oxides	%	Oxides	%
CuO	49	SO_3_	0.43
ZnO	32.2	K_2_O	0.07
Al_2_O_3_	13.9	MgO	0.09
Fe_2_O_3_	0.29	P_2_O_5_	0.02
CaO	0.59	Others (NiO, ZrO₂, Ta₂O₅, Cl^−^)	0.17
SiO_2_	0.2	Loss on Ignition (LOI)	2.97

Phosphate adsorption is highly facilitated by the two main components, CuO and ZnO, through various mechanisms because of their dominant presence:

1. *Surface hydroxyl group formation*: When applied to an aqueous environment, both CuO and ZnO develop surface hydroxyl groups (-OH) through hydration reactions. Phosphate ions mainly bind to these hydroxyl groups, which are active sites, through ligand exchange reactions, whereby a phosphate ion substitutes a surface hydroxyl group, and hence a stable inner-sphere complex is formed.
2. *Amphoteric behavior*: Zinc oxide (ZnO) is amphoteric and works well over a wide pH range (pH 6–10). At the pH of typical wastewater (7–8), both positively and negatively charged zinc hydroxide surface species are formed, which thus gain the ability to attract phosphate anions electrostatically^54^.
3. *Surface complexation capability*: At the oxide–water interface, Cu^2+^ and Zn^2+^ ions can form multi-dentate complexes with phosphate via coordination bonds. Some spectroscopic techniques, such as FTIR, demonstrated that copper and zinc make chemical bonds respectively with oxygen and phosphate atoms, thus indicating chemisorption^54^.
4. *Aluminum oxide contribution*: Despite its small amount of 13.9% only, aluminum oxide has a major role in binding phosphate as alumina is famous as a material for phosphate removal due to the high affinity of this material towards phosphate, attributed to the acid base interaction of Al^3+^ sites with phosphate. Aluminum-phosphate interaction gives rise to the formation of precipitated AlPO₄ (as shown in the XRD results).
5. Other minor constituents such as Fe_2_O_3_, CaO, which may also be found, provide more adsorption surfaces. Phosphates exhibit strong affinity for iron oxide surfaces, where the predominant bonding mechanism is ligand exchange^55^. However, calcium can help in the precipitation process for creating calcium phosphate minerals under favorable circumstances. Although silica dioxide is known for its inactive nature, it can still make an indirect contribution through the improvement of structural stability and dispersion of the active phases. The low loss on ignition percentage (2.97%) indicates that the sample has no volatile content and is stable at high temperatures, making it ideal for situations where the regeneration process involves heat treatment. The presence of sulfur (0.43% SO_3_) is crucial, as it indicates the residual sulfur substances that have been accumulating during industrial operations. Such a discovery suggests that the catalyst might be poisoned, which is a common deactivation mechanism associated with LTS catalysts due to sulfur substances attaching themselves to the active metal sites, thereby reducing catalytic activity. However, from the perspective of adsorption, such residual substances can influence the adsorption behavior.

#### Mineralogical analysis (XRD analysis)

**Fig. 1:**
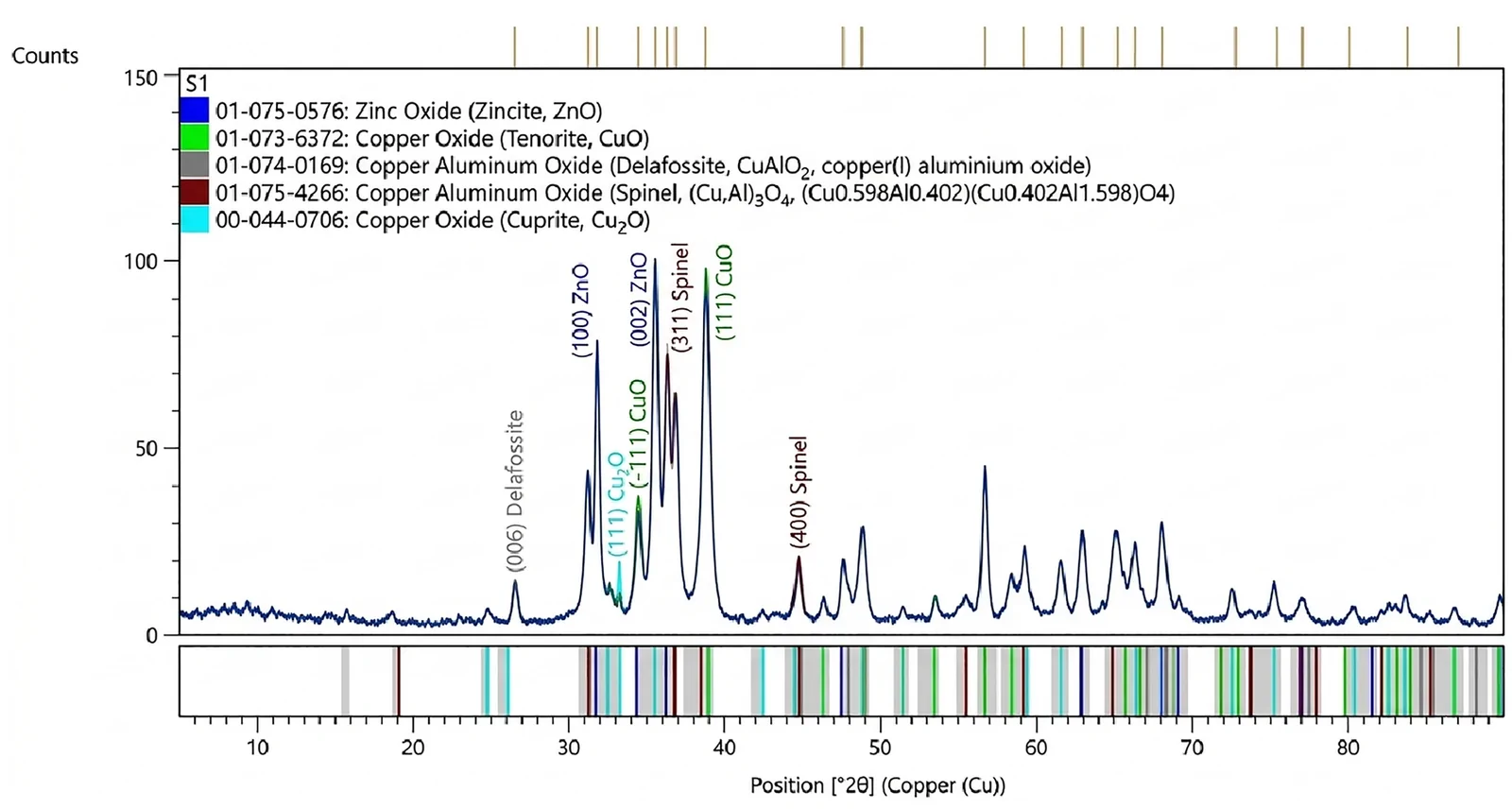
Mineralogical analysis of LTSSC before phosphate ion adsorption.

**Fig. 2:**
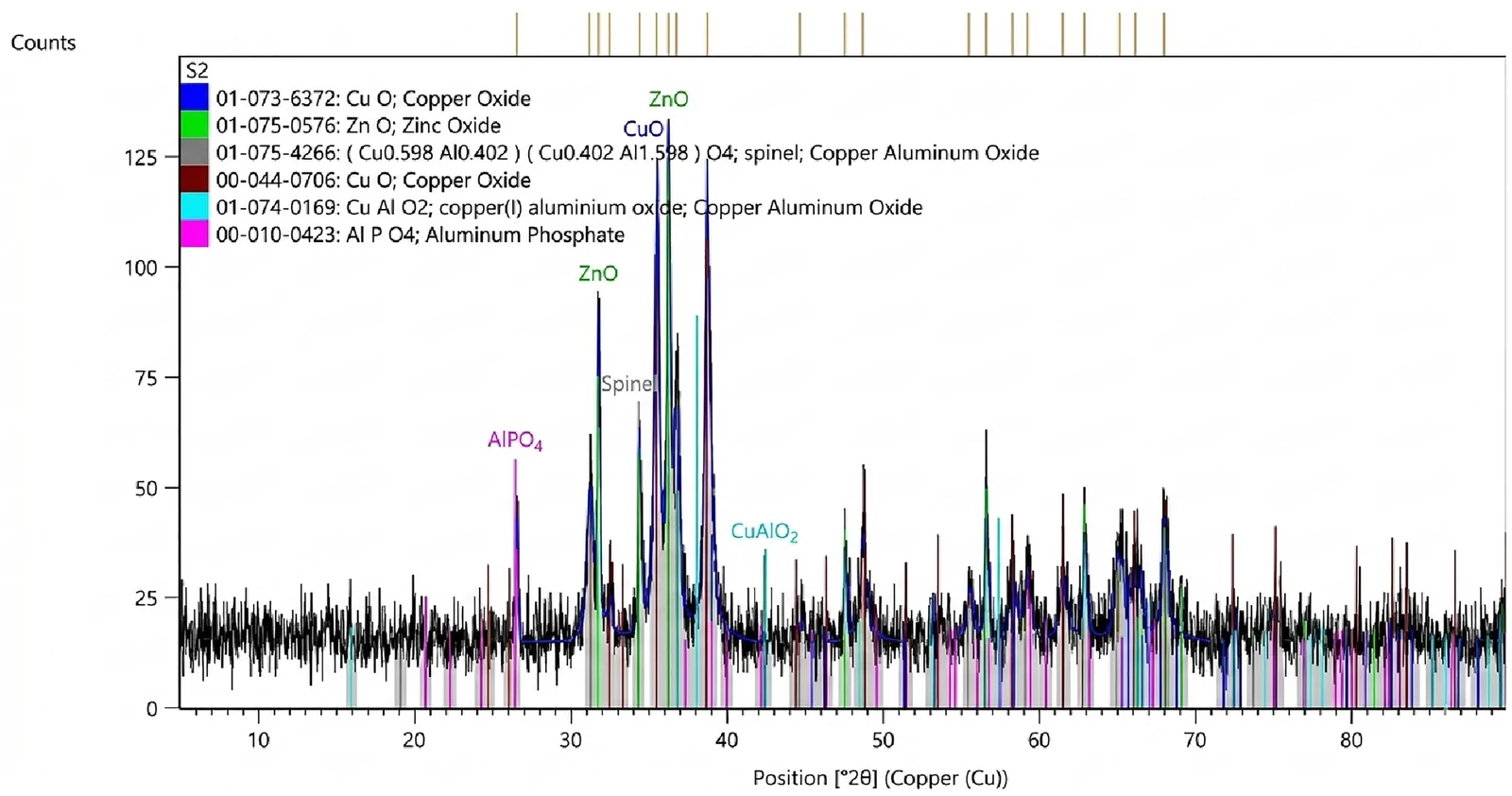
Mineralogical analysis of LTSSC after phosphate ion adsorption.

Figures 1 and 2, respectively, illustrate the XRD patterns of LTSSC before and after phosphate adsorption. Identification of the phases using the ICDD database indicates the crystalline phases existing and their structural changes during adsorption.

##### Fresh LTSSC (before adsorption)

The XRD pattern shows characteristic diffraction peaks of four main crystalline phases:

1. Zinc oxide (ZnO—Zincite, PDF #36–1451): The sharp diffraction peaks at 2θ = 31.8°, 34.4°, 36.3°, 47.5°, 56.6°, 62.9°, 66.4°, 68.0°, and 69.1° correspond to (100), (002), (101), (102), (110), (103), (200), (112), and (201) planes of the hexagonal wurtzite. The high peak intensity and narrow full width at half-maximum (FWHM) demonstrate that ZnO is well crystallized, and the crystallite size is estimated to be 35–40 nm as calculated using the Scherrer formula^54^.
2. Copper(II) oxide (CuO—Tenorite, PDF #48–1548): The major peaks at 2θ = 35.5°, 38.7°, 48.7°, 53.5°, 58.3°, 61.5°, 66.2°, 68.1°, and 72.4° are related to (002), (111), (202), (020), (202), (113), (311), (220), and (311) diffraction planes of monoclinic CuO structure. The crystallite size is about 25–30 nm^56^.
3. Copper aluminum oxide (CuAlO₂—Delafossite, PDF #35–1401): The diffraction peaks at 2θ = 31.7°, 36.8°, 42.3°, and 61.3° correspond to (006), (101), (012), and (110) planes of the rhombohedral delafossite structure. This mixed metal oxide phase is formed when CuO reacts with Al₂O₃ during catalyst synthesis at high temperature^57^.
4. Copper aluminum spinel (Cu₀.₅₉₈Al₀.₄₀₂O₄, PDF #78–1605): Weak diffraction peaks at 2θ = 19.0°, 31.3°, 36.9°, 44.8°, and 55.7° correspond to the cubic spinel structure. Spinel phases are widely known to have high stability, porosity, and cation exchange capacity, which are beneficial features for adsorption applications^57^.

Rietveld refinement quantitative phase analysis results in ZnO: 42 ± 3 wt%, CuO: 38 ± 3 wt%, CuAlO₂: 15 ± 2 wt%, and Cu-Al spinel: 5 ± 1 wt%. These figures correspond well with XRF elemental analysis when differences between oxide and elemental weights are taken into consideration.

##### LTSSC after phosphate adsorption

Post-adsorption XRD shows an important change in the structure: the discovery of a new crystalline phase.

Aluminum phosphate (AlPO₄—Berlinite, PDF #76–1782): Additional peaks can be noticed at 2θ = 20.8°, 23.4°, 26.6°, 29.7°, 36.6°, and 50.5°, which correspond to (100), (101), (110), (111), (202), and (212) reflections of the hexagonal AlPO₄ structure that is structurally similar to α-quartz^58^. The detection of crystalline AlPO₄ serves as strong evidence for a chemical reaction between aluminum-containing phases (Al₂O₃, CuAlO₂, Cu-Al spinel) and phosphate ions. The mechanism of formation is most probably:

1. Surface complexation: In the beginning, phosphate gets attached to the surface Al–OH groups through ligand exchange (≡Al–OH + H₂PO₄^−^ → ≡Al-H₂PO₄ + OH^−^ & ≡Al–OH + HPO₄^2^^−^ → ≡Al-HPO₄^−^ + OH^−^)
2. Surface precipitation: When the local phosphate concentration at the adsorbent surface is high, aluminum ions are slightly dissolved (especially at pH < 9) and react with phosphate to generate an insoluble precipitate of AlPO₄ (Al^3+^ + PO₄^3−^ → AlPO₄↓ (Ksp = 9.84 × 10^−21^))

The fact that ZnO and CuO peaks are still there without any changes in intensity or positions is an indication that these phases mainly contribute to the adsorption process through their surface (complexation, electrostatic attraction) rather than bulk precipitation. In other words, they behave as less reactive species than aluminum oxide at the experimental pH level (pH 8).

#### Surface area and pore structure (BET analysis)

**Fig. 3:**
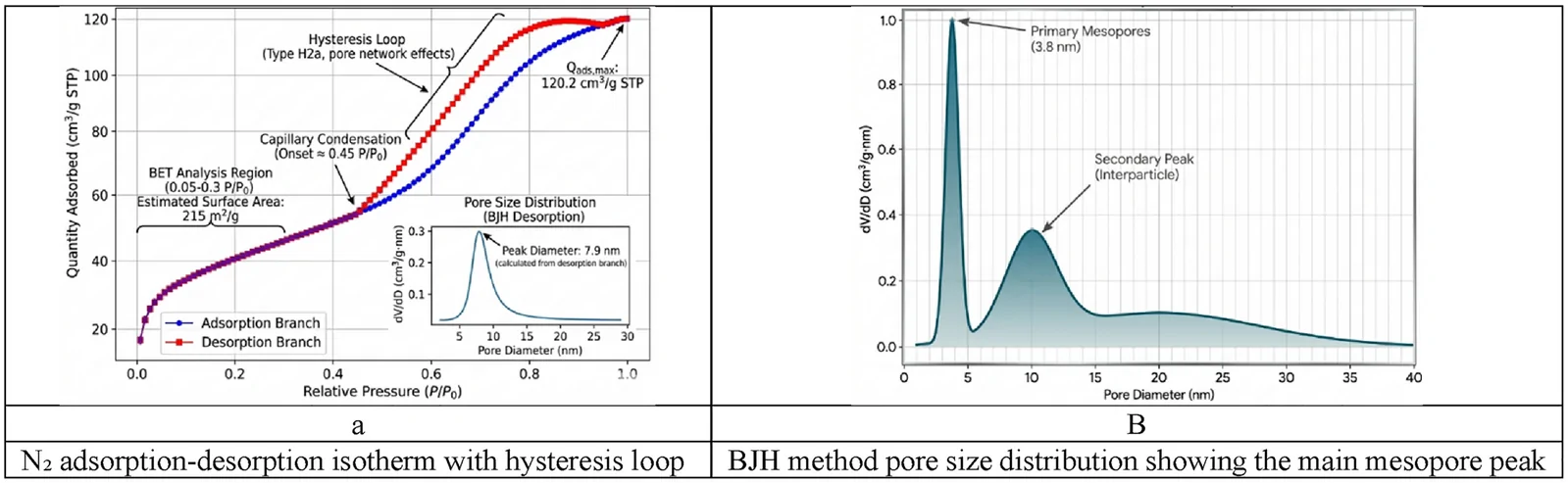
BET Resulted Figures for LTSSC.

**Fig. 4:**
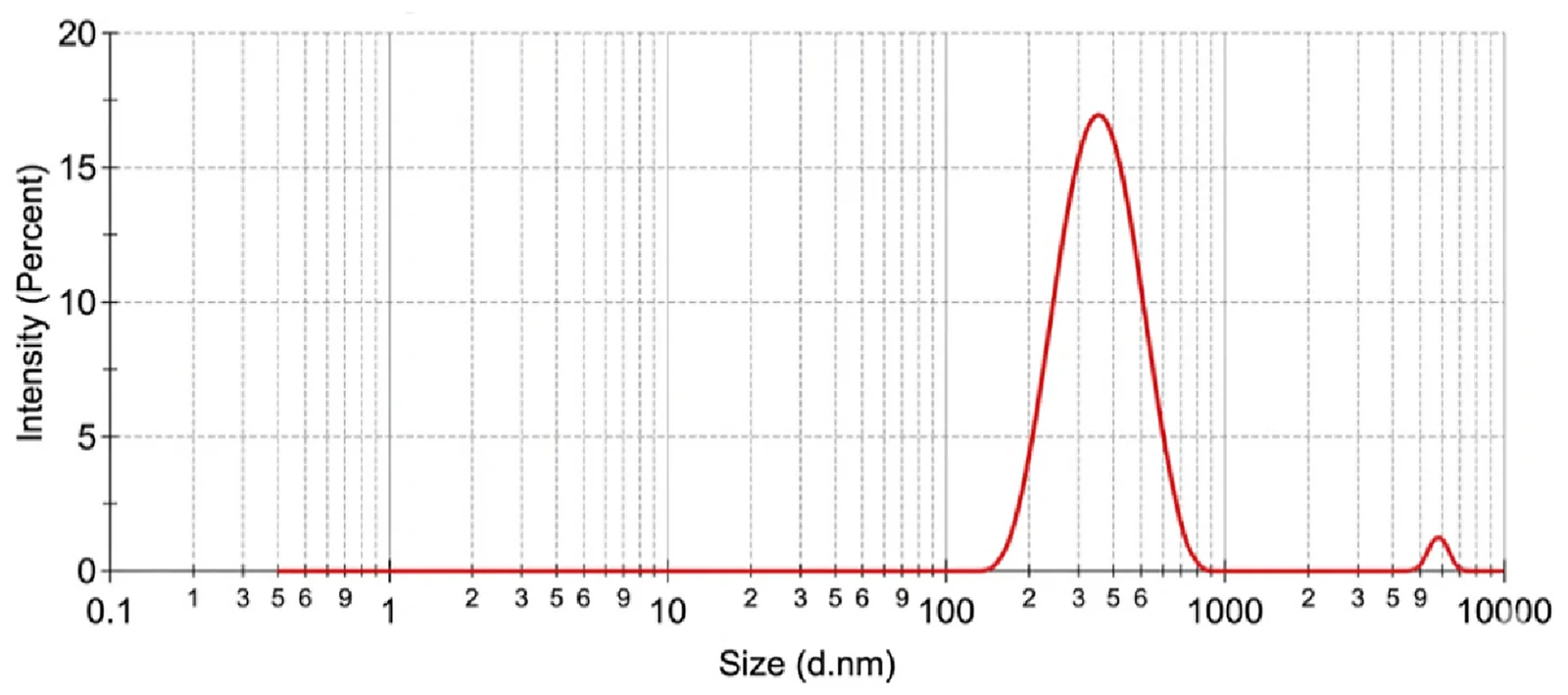
Particle size distribution of LTSSC.

Nitrogen adsorption–desorption isotherms and pore size distribution of LTSSC are presented in Figs. 3 and 4, respectively. BET surface area analysis reveals critical textural properties governing adsorption performance. The results of the BET Analysis are shown in Table 5.

**Table 5:** Key surface area parameters.

Parameter	Value
BET surface area	247 ± 5 m^2^/g
Total pore volume (at P/P₀ = 0.99)	0.185 cm^3^/g
Micropore volume (t-plot)	0.038 cm^3^/g (20.5% of total)
Mesopore volume	0.147 cm^3^/g (79.5% of total)
Average pore diameter	3.8 nm
External surface area (t-plot)	198 m^2^/g

The N₂ adsorption–desorption isotherm in Fig. 3a, according to the IUPAC classification, signifies a predominantly mesoporous structure. The isotherm exhibits a sharp nitrogen uptake at low relative pressure (P/P₀ < 0.1), which is explained by the filling of the micropores and represents roughly 20 percent of the total pore volume. This is followed by a slow increase at the medium pressure range (P/P₀ = 0.1–0.8) as a result of multilayer adsorption on mesopore walls. The sudden rise in adsorption at the relatively high pressures (P/P₀ = 0.4–0.9) is consistent with capillary condensation inside a mesopore with a size in the 2–50 nm range. The occurrence of an H3-type hysteresis loop is indicative of the differences between the adsorption and desorption branches that reflect slit-shaped pores or plate-like particle aggregates with non-uniform pore sizes, which is consistent with the SEM results of irregular and aggregated particle morphology.

The pore size distribution presented in Fig. 3b, which is a result of the BJH analysis of the adsorption branch, shows that the mesoporous fraction dominates the pores at around 3.5–4.2 nm, which is indicative of a highly developed mesoporous structure. There is another, smaller peak at 8–12 nm, which is interpreted as larger mesopores originating from interparticle spaces. The rest of the pore volume is quite broadly distributed up to 30 nm, and this is considered to reflect the textural heterogeneity of the material.

Quantitative analysis shows mesopores make up about 87% of the total pore volume, while macropores and micropores make up 11% and 2%, respectively. This type of pore structure is very advantageous for adsorption purposes, as mesopores (2, 50 nm) allow for easy diffusion and access of the phosphate ions. The small micropore part means there are very few pore blockage effects; on the other hand, the existence of macropores leads to improvement of mass transfer because they serve as transport channels. Considering the hydrated ionic radius of phosphate species (~ 0.5 nm), the main mesoporous structure offers more than enough surface area for the reactant species, so there will be hardly any steric hindrance during adsorption. The BET surface area of LTSSC (247 ± 5 m^2^/g) is lower than that of the fresh catalyst (~ 350 m^2^/g), which can probably be explained by thermal sintering and structural collapse during very long industrial operation. On the other hand, the high surface area that is still retained and the good pore structure allow the effective adsorption performance to be carried out.

Importance of phosphate adsorption:

1. High surface area (247 ± 5 m^2^/g): Gives many active sites for phosphate binding. The measured specific surface area is much higher than most agricultural waste-derived adsorbents (typically 50–150 m^2^/g)^59^ and is at the level of some commercial activated carbons, thus unnecessarily having a high adsorption capacity despite quite low q_max_ (0.174 ± 0.005 mg/g), being due to specific phosphate-metal oxide interactions.
2. Predominance of mesoporous structure: Pore diameters in the range of 3–10 nm are considered ideal for phosphate ion accessibility (phosphate ionic diameter ~ 0.3–0.5 nm). Mesopores not only allow for the fast diffusion of phosphate inside the material but also provide enough surface area for the binding process. Micropores (< 2 nm), though adding to the surface area, may have to face the limitation of phosphate ion diffusion.
3. Hierarchical pore structure: The synergy of micropores (giving very high surface area), small mesopores (providing active adsorption sites), and large mesopores (acting as transport channels) results in an effective mass transfer network, thus the relatively fast adsorption kinetics found from the experiment (equilibrium within 4 h) can be explained.

Comparing the two after thermal activation, the non-activated spent catalyst showed a specific surface area of 189 m^2^/g, whereas thermal treatment at 400 °C further increased the surface area to 247 ± 5 m^2^/g, which is a 31 percent improvement. This increase is a result of the removal of residual carbon deposits and process oils that blocked the entrances of pores, the thermal decomposition of hydroxides and carbonates into oxide phases, which have higher intrinsic porosity, and partial sintering that leads to the formation of new interconnected pore channels inside the catalyst structure.

Following phosphate adsorption, the BET analysis shows that the surface area reduced to 201 ± 5 m^2^/g, thus supporting the conclusion that the pores were occupied by phosphate species. Pore volume dropped from 0.185 to 0.151 cm^3^/g, indicating ~ 18% pore filling, which is in line with the adsorption capacity that was observed and implies that there might be some capacity left for further optimization.

#### Particle size distribution

Analysis by laser diffraction (Fig. 4) shows the particle size distribution of the LTSSC after grinding and sieving. The distribution curve is typical of a single peak (monomodal); however, the peak is not symmetric and tails toward the larger particles (Table 6).

**Table 6:** Key particle size parameters.

Parameter	Value
D₁₀ (10% cumulative undersize)	201 ± 18 nm
D₅₀ (median diameter)	401 ± 20 nm
D₉₀ (90% cumulative undersize)	823 ± 10 nm
Mean diameter (volume-weighted)	448 ± 15 nm

Particle size distribution is directly related to the performance of adsorption. The submicron average particle size of 401 ± 20 nm significantly contributes to the enhancement of surface area since the surface area and particle diameter are inversely related, which elucidates the determined BET surface area of 247 ± 5 m^2^/g in the case of porous mixed metal oxide particles having an average density close to 5.5 g/cm^3^. Besides this, the diminutive particle size enhances the rate of mass transfer by the reduction of the length of internal diffusion paths; thus, phosphate runs faster to active binding sites, explaining the rapid adsorption equilibrium (within 4 h).

#### Surface morphology (scanning electron microscope (SEM))

**Fig. 5:**
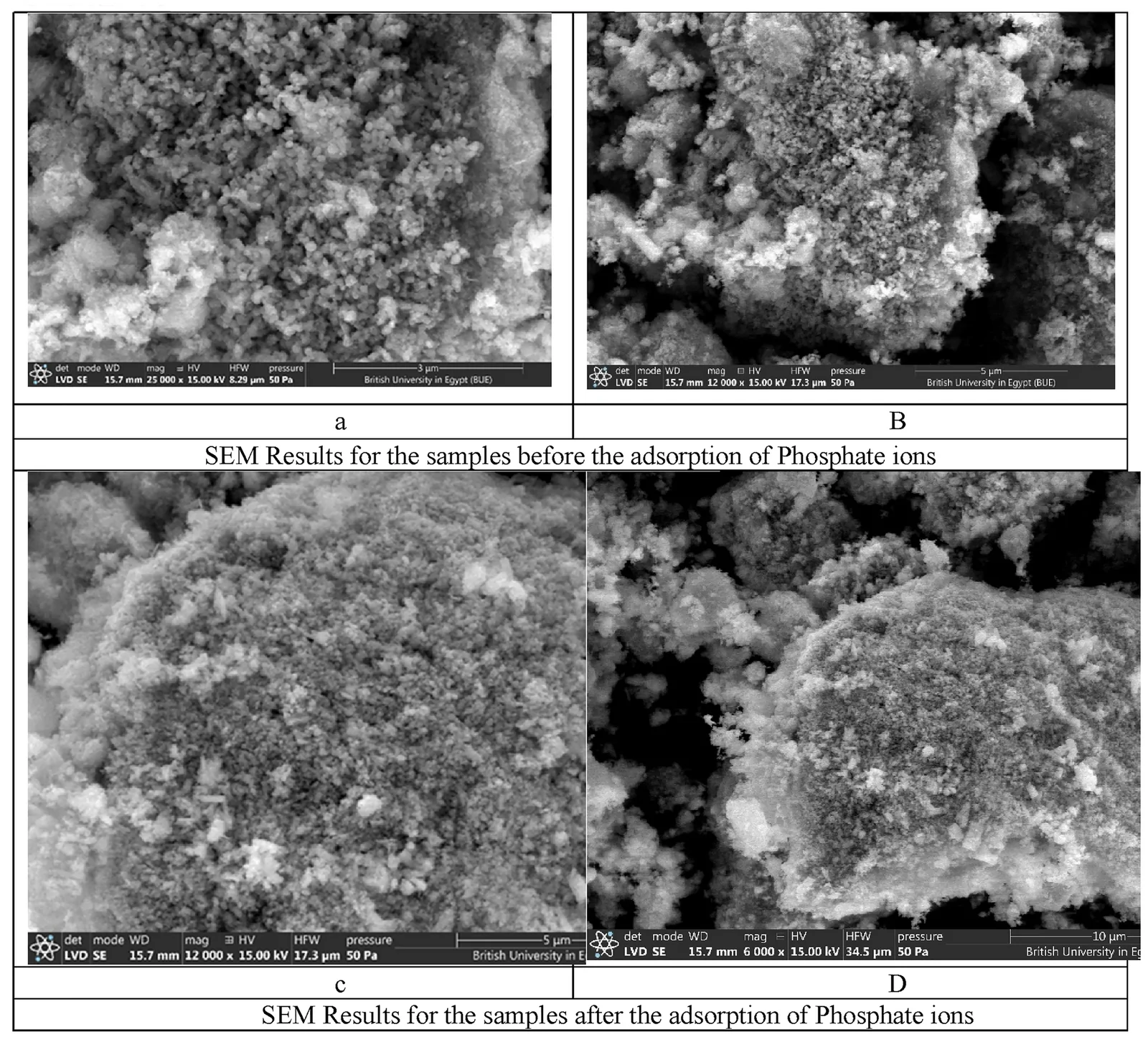
SEM images—(**a**, **b**) before adsorption showing porous structure; (**c**, **d**) after adsorption showing surface coverage and deposits.

The electron scanning microscope technique provides images displaying the surface morphology of the adsorbent before and after the adsorption occurs^60^. Hence, Fig. 5 illustrates the structural modifications of the LTSSC surface during the phosphate adsorption process.

##### Before phosphate adsorption

SEM observations reveal that at 500–2,000 × magnification, the LTSSC powder is made of irregular agglomerates of 5–50 μm size range, which are formed by aggregation of primary nanoparticles, a feature typical for mechanically ground metal oxide catalysts and attributed to strong van der Waals and electrostatic attractions between high surface area particles. At further magnifications of 10,000–30,000 × , the pictures show a rough and heterogeneous surface with a lot of protrusions and indentations as well as cracks and fissures that give rise to macropore channels larger than 50 nm and thus facilitate the penetration of fluids to the inside of the particle. A sponge-like porous microstructure can be seen that corresponds to the meso-porosity detected by BET analysis, and the measured primary crystallite size of 200–800 nm is in good agreement with laser diffraction data. These surface irregularities and defect sites serve as adsorptive centers with high energy, and the rough, porous morphology with a high surface-to-volume ratio in general accounts for the relatively high phosphate adsorption capacity.

##### After phosphate adsorption

The surface morphology of LTSSC changed significantly, as evidenced by SEM microscopy. Initially, the rough surfaces of LTSSC become partially covered with smoother, granular deposits, which are assigned to absorbed phosphate species and newly formed aluminum phosphate. Besides, XRD analysis unambiguously identifies aluminum phosphate (AlPO₄) as the new compound. The particle aggregation degree increases substantially as phosphate species act as molecular bridges between particles. Phosphate bridges are formed by three mechanisms: hydrogen bonding, multidentate phosphate metal complexes, and surface charge neutralization near the isoelectric point. At this point, electrostatic repulsion is diminished and, thus, more compact, densely packed agglomerates are generated than those before adsorption. Small surface pores in the 2–10 nm range are observed to be partially or fully filled, and bright areas reveal the presence of phosphorus-rich zones while deposition on the surface decreases the size of pore openings and provides a satisfying explanation of the decrease of BET surface area from 247 ± 5 to 201 ± 8 m^2^/g after adsorption. New surface phases are seen in the form of isolated, angular crystallites having a size of 100–500 nm that correspond to AlPO₄ precipitates revealed by XRD. Eventually, the surface pattern changes from irregular and porous to relatively smoother and more continuous; however, total pore blockage does not happen, and thus about 60–70 percent of the original surface area is still accessible, which, in turn, is indicative of favorable regeneration and reuse possibilities after a single adsorption cycle.

#### Energy dispersive X-ray (EDX)

Energy-dispersive X-ray spectroscopy determines the amounts of the elements present and where they are located on the surfaces of LTSSC. It thus offers the most direct indication of phosphate adsorption on a small scale^51^.

##### Before phosphate adsorption

The EDX spectrum shows typical elemental peaks that reveal the material composition. Copper is confirmed as the major component by three strong signals at Cu Lα (0.93 keV), Cu Kα (8.04 keV), and Cu Kβ (8.91 keV), while the presence of zinc is suggested by the peaks at Zn Lα (1.01 keV), Zn Kα (8.64 keV), and Zn Kβ (9.57 keV). Aluminum exists in the sample, but its signal intensity is very low, which is less than that for copper and zinc; the oxygen signal at O Kα (0.52 keV) indicates the formation of oxides. The results of EDX before adsorption are shown in Table 7.

**Table 7:** Quantitative EDX analysis (normalized to 100%, excluding carbon and hydrogen).

Element	Atomic %	Weight %
O	52.3	35.1
Cu	18.7	49.8
Zn	15.2	41.7
Al	13.8	15.6

The comparison of these values with the XRF oxide composition data (CuO 49%, ZnO 32.2%, Al₂O₃ 13.9%) indicates the validity of the multi-technique characterization approach. The little excess in oxygen atomic percentage may result from the fact that metals are in their fully oxidized state, plus there are some surface hydroxyl groups.

##### After phosphate adsorption

Phosphorus detection shows a significant transformation with the appearance of two distinct phosphorus peaks. These are a small PLα peak at 0.21 keV and a large, well-resolved PKα peak at 2.01 keV. That offers clear proof of phosphate adsorption onto the adsorbent surface. Table 8 shows EDX results after adsorption.

**Table 8:** Quantitative analysis post-adsorption.

Element	Atomic %	Weight %
O	55.8	38.2
Cu	16.9	46.0
Zn	13.7	38.3
Al	11.2	12.9
**P**	**2.4**	**3.2**

##### Key observations

**Fig. 6:**
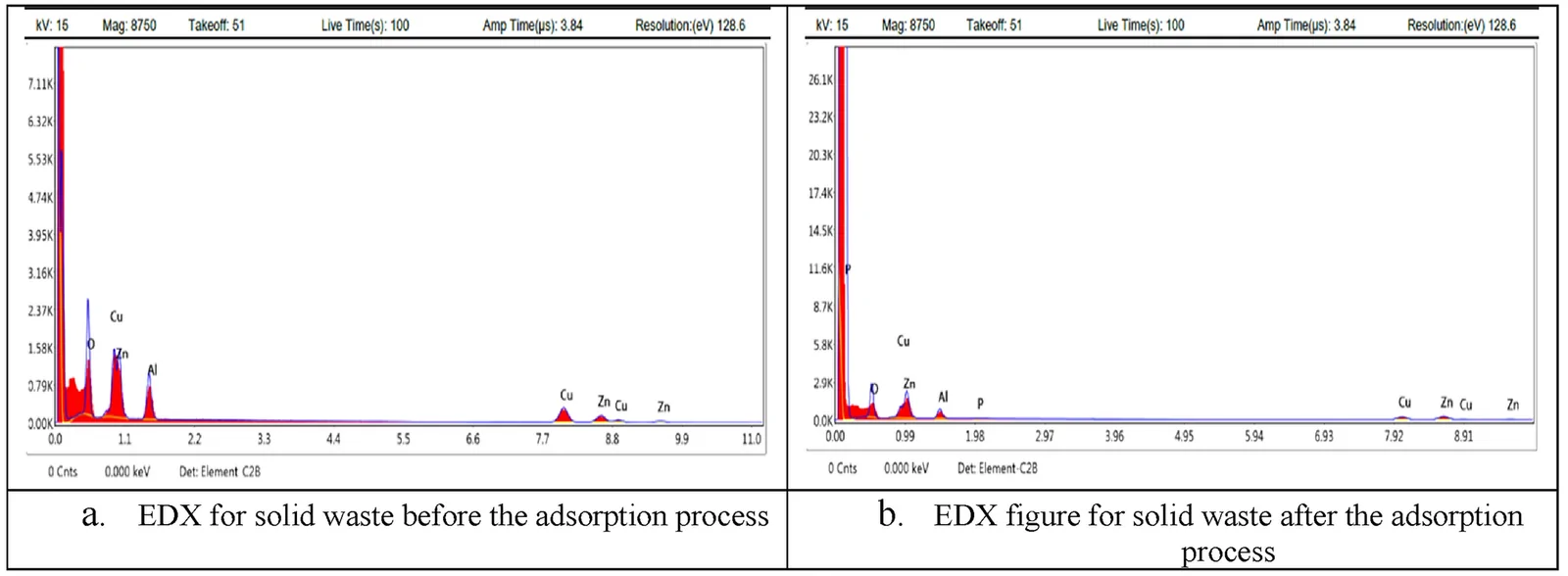
EDX spectra comparison—(**a**) before adsorption showing no P peaks; (**b**) after adsorption with clear P peaks.

EDX analysis indicates phosphorus incorporation, with 2.4 atomic% (3.2 wt%) phosphorus detected across the analyzed surface regions. The higher phosphorous content in the surface-layer material can be seen in this surface weighting analysis. At the same time, there is an increase in the atomic percentage of oxygen from 52.3% to 55.8%. This can be explained by the oxygen content of phosphates (where each PO₄^3−^ ion consists of four oxygen atoms). There is a small reduction in the atomic percentage of Cu, Zn, and Al. This is not due to the depletion of metals but is due to their dilution with phosphorus (Fig. 6).

#### Functional group analysis Fourier transform infrared (FTIR spectroscopy)

**Fig. 7:**
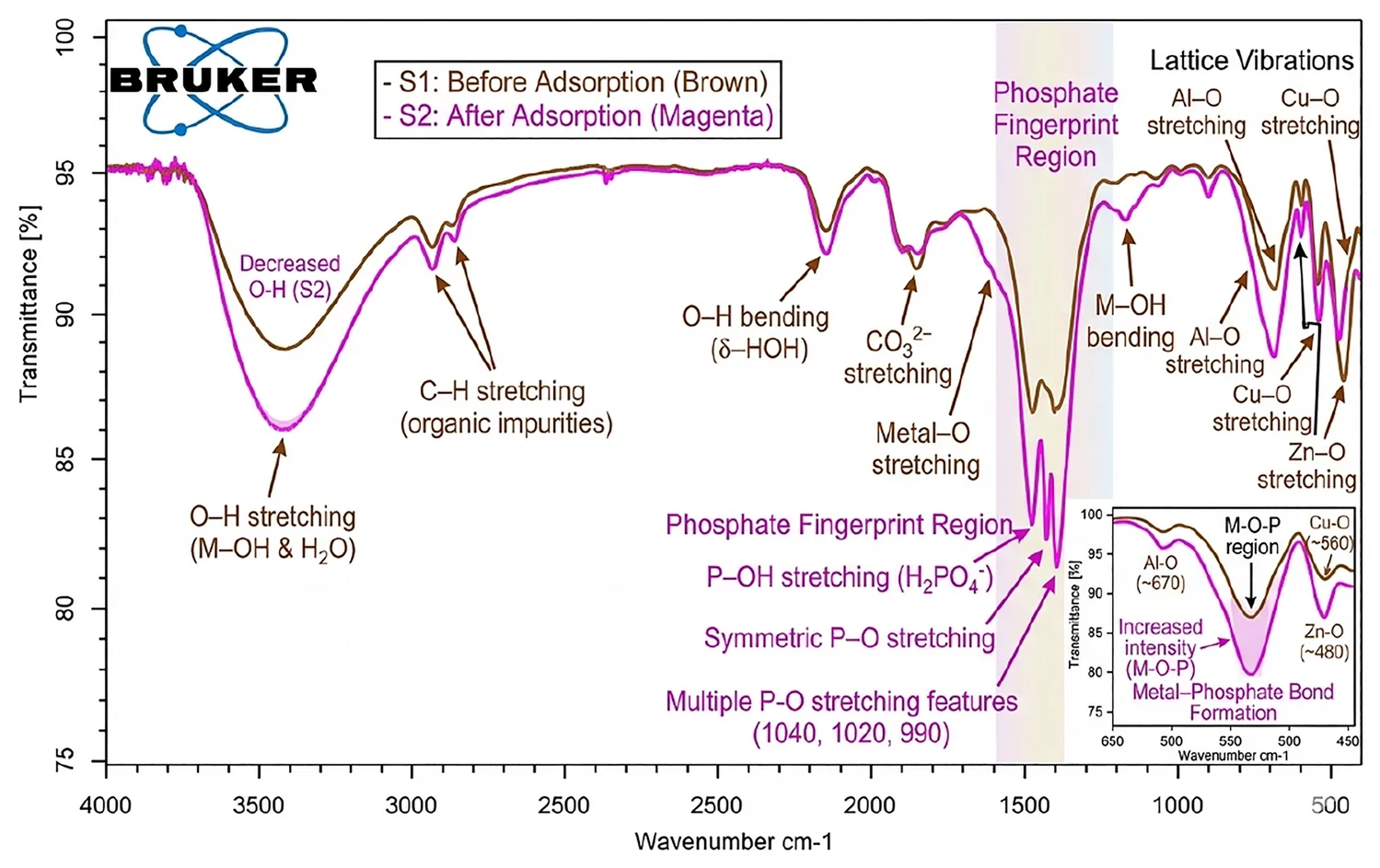
FTIR spectra overlay showing S1 (before) and S2 (after) with highlighted phosphate region 900–1200 cm^−1^.

Fourier-transform infrared spectroscopy (FTIR) is a technique that helps us know the types of chemical groups on the surface that are the main reasons for binding phosphates, and it also indicates that chemical interactions indeed occur during adsorption. We can see the FTIR spectra of the samples before (S1) and after (S2) phosphate adsorption in Fig. 7.

##### Spectrum before phosphate adsorption

A broad, intense band at 3200–3600 cm^−1^ centered around 3420 cm^−1^ corresponds to O–H stretching vibrations from surface hydroxyl groups (M–OH, where M = Cu, Zn, and Al) and adsorbed water molecules. Its high intensity indicates an abundance of surface hydroxyls that are essential for phosphate binding via a ligand-exchange mechanism. The C–H stretching vibrations from residual organic impurities, such as process lubricants or hydrocarbons from ammonia synthesis feed, are responsible for the weak bands at 2920 and 2850 cm^−1^. Their low intensity attests to efficient washing and minimal organic interference. While weak shoulder features in the 1380–1450 cm^−1^ range are attributed to asymmetric stretching of carbonate (CO₃^2−^), indicating minor air-exposure contamination, a medium-intensity band at ~ 1630 cm^−1^ may arise from O–H bending (δ-HOH) of adsorbed water and/or carbonate species created by atmospheric CO₂ interaction with basic metal oxide surfaces. Metal–O stretching vibrations typical of metal oxide lattices are represented by weak, broad bands in the 1100–1200 cm^−1^ area, while M–OH bending vibrations are linked to weak features at 900–1000 cm^−1^. Lattice vibrations are responsible for strong multiple peaks in the 400–800 cm^−1^ area, such as Al–O stretching at ~ 670 cm^−1^ in Al₂O₃ and aluminum-containing phases, Cu–O stretching at ~ 560 cm^−1^ in the CuO lattice, and Zn–O stretching at ~ 480 cm^−1^ in the ZnO lattice.

##### Spectrum after phosphate adsorption

The 900–1200 cm^−1^ range corresponds to the fingerprint region of the phosphate family and exhibits spectral shifts due to the emergence of bands characteristic of phosphate species: an additional faint band in the range of 870–920 cm^−1^, which corresponds to P-OH stretching of the protonated phosphate ions (H₂PO₄^−^ and HPO₄^2−^), existing simultaneously at pH 8 as per phosphate species; a broadening of the peak in the range of 990–1020 cm^−1^, due to symmetric P-O stretching, shifted towards higher wavenumbers as a result of interaction with metal sites in S2. Furthermore, higher M–O–P vibrations (M = Cu, Zn, and Al) are linked to increased intensity in the 540–600 cm^−1^ area, directly demonstrating metal–phosphate bond formation through frequency shifts and intensity increases. The broad O–H stretching band at 3200–3600 cm^−1^ has decreased and narrowed, indicating the consumption of surface hydroxyl groups through ligand exchange with phosphate (εM–OH + H₂PO₄^−^ → ≡M–OPO₃H^−^ + H₂O). The band at ~ 1630 cm^−1^ has remained mostly unaltered, suggesting the maintenance of the hydration layer associated with adsorbed phosphate.

FTIR evidence indicates that phosphate adsorption proceeds primarily via a ligand-exchange mechanism. Inner-sphere complexation, in which phosphate directly coordinates to metal centers without the intervention of water molecules, is further indicated by the observed shift of P–O stretching frequencies from those of free phosphate (960–980 cm^−1^) to higher wavenumbers (1040–1100 cm^−1^). This strong chemical interaction explains the pseudo-second-order kinetic behavior and high adsorption affinity. The existence of different bonding sites as monodentate, bidentate, and bridging sites can be attributed to the multiple peaks in the P-O stretching region corresponding to 1040, 1020, and 990 cm^−1^. The adsorption energies are distributed unevenly, which is reflected in the good fit for the Freundlich isotherm equation. The adsorbed phosphate still exhibits partial protonation in the form of H₂PO₄^−^ and HPO₄^2−^ but not completely deprotonated into PO₄^3−^ due to the appearance of the weak P-OH band at 870–920 cm^−1^. It reflects the phosphate species existing in a ratio of 1:4 at pH 8. Moreover, there are no bands in the range of 960–980 cm^−1^ indicating the existence of physically adsorbed phosphate species in the form of free or electrostatically bound species.

#### Zeta potential

**Fig. 8:**
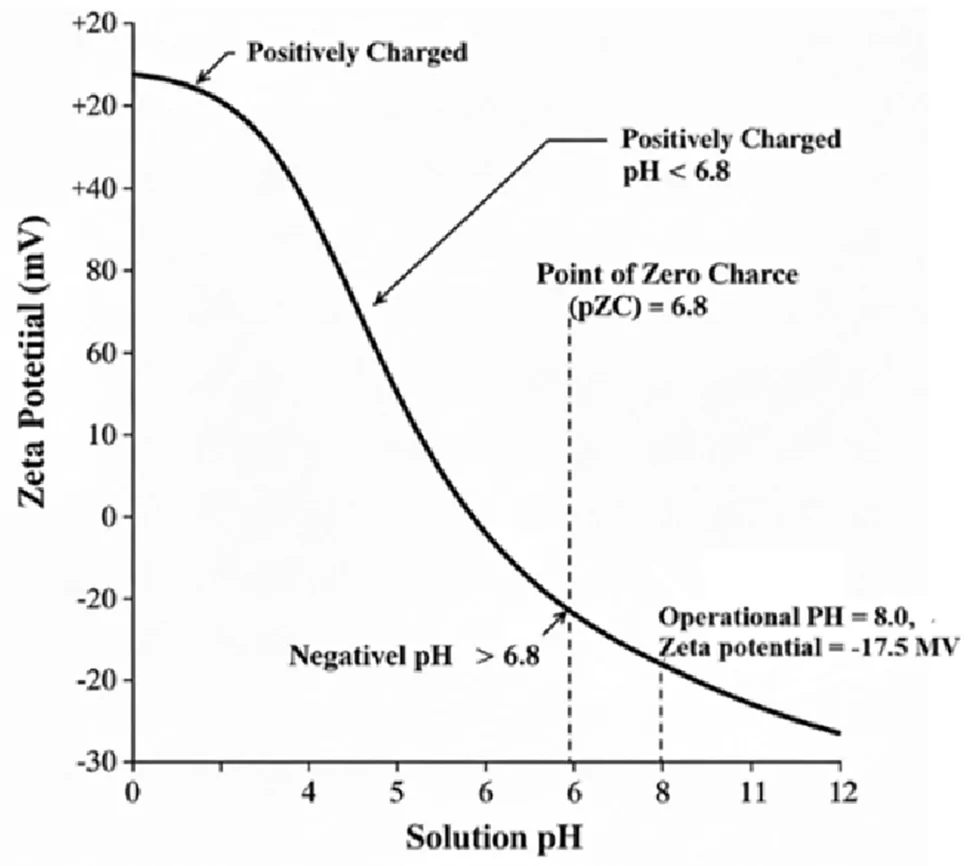
Zeta potential vs. pH curve.

Determining the point of zero charge (pZC) and zeta potential as a function of pH is essential for comprehending the electrostatic interactions between the phosphate anions and the LTSSC surface. LTSSC displays a pH dependent surface charge typical of amphoteric metal oxides: at pH 2–4, protonation (M–OH + H^+^ → M–OH^2+^) causes the surface to be positively charged with a zeta potential of + 15 to + 8 mV; between pH 5–6, the surface approaches a near-neutral state near the point of zero charge (pZC), which is measured at pH 6.8 where the zeta potential is 0 mV. Deprotonation (M–OH + OH^−^ → M–O^−^ + H₂O) causes the surface to become negatively charged at higher pH values of 7–11, with zeta potentials ranging from − 5 to − 22 mV. LTSSC displays a moderately negative zeta potential of − 17.5 mV at the experimental pH of 8.0, as shown in Fig. 8.

##### Significance for phosphate adsorption

The LTSSC adsorbent surface and phosphate species are both negatively charged at pH 8. However, based on the Henderson-Hasselbalch equation (pKa₂ = 7.2), phosphate ions are mainly in the form of HPO₄^2−^ (79%) and H₂PO₄^−^ (21%), while the zeta potential for surface M–O^−^ groups is − 17.5 mV, leading to a supposed electrostatic repulsion contradiction. Phosphate ions bond with metal ions through covalent M–O-P bonds, in line with the effective adsorption capacity above pZC, pseudo-second order kinetics, and FTIR analysis demonstrating covalent bonding. However, despite the strong electrostatic repulsion, adsorption of phosphate ions occurs efficiently (92.95 ± 2.8%). Phosphate removal occurs at its maximum rate between the pH range 7 to 9, where the pZC is slightly higher than 7 according to the optimization results. At lower pH levels compared to the pZC, there is reduced selectivity due to competitive adsorption with other anions (such as Cl^−^ and SO₄^2−^) and fewer M-OH groups on the surface despite electrostatic interactions favoring adsorption; however, at higher pH values (> 10), high surface negativity and competing hydroxide ions hinder phosphate adsorption. This synergistic interaction of CuO (with pZC of ~ 9.5), ZnO (pZC of ~ 9.0) and Al₂O₃ (pZC of ~ 8.5) is evident in the composite value of pZC equal to 6.8. At a pH level of 8, the surfaces of CuO and ZnO are less negatively charged than those of Al₂O₃^59^

#### Thermal analysis (TGA-DTG)

**Fig. 9:**
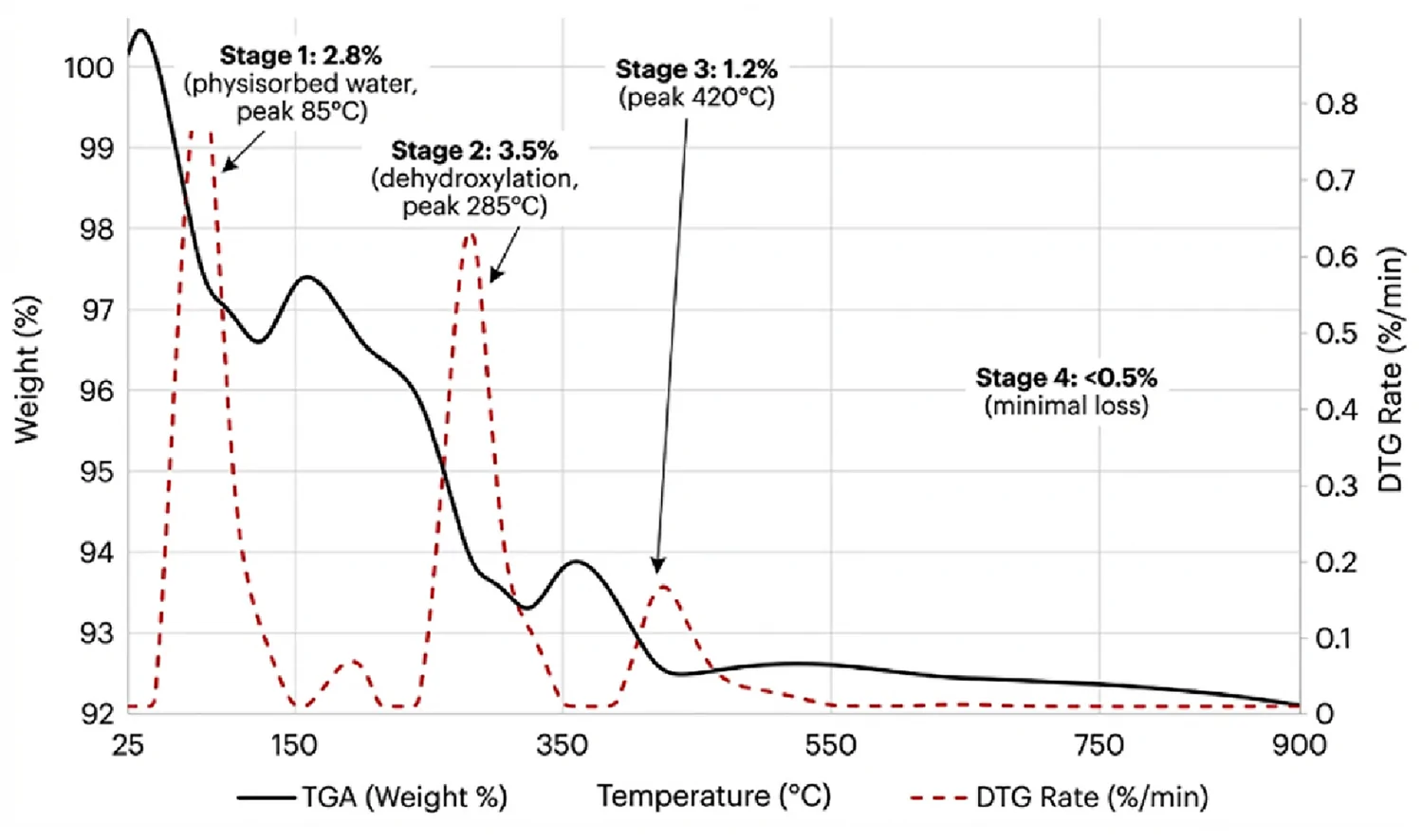
TGA-DTG curves showing weight loss stages and corresponding temperature ranges.

The best regeneration temperature is chosen based on the thermal stability, breakdown behavior, and weight loss features of LTSSC that are revealed by thermogravimetric analysis, as shown in Fig. 9.

##### TGA curve (weight % vs. temperature)

Four separate phases of weight loss throughout a temperature range of 25 to 900 °C, totaling 8.0%, are shown by thermogravimetric analysis of LTSSC. Physisorbed water and surface moisture cause 2.8% of the weight loss in Stage 1 (25–150 °C), with a large endothermic DTG peak at 85 °C. Stage 2 (150 – 350 °C): DTG peak appears at 285 °C, and there is a 3.5% weight loss caused by the dehydroxylation reaction of hydroxyl groups on the surface along with the decomposition of any organic contaminants left behind (2 M-OH → M–O-M + H2O). Stage 3 (350 – 550 °C): There is a minor DTG peak observed at 420 °C and a 1.2% weight loss caused by further dehydroxylation reactions and the release of structure-bound water molecules from hydrates. Stage 4 (550 – 900 °C): Very minimal weight loss (less than 0.5%) occurs due to the stability of the metal oxides (CuO, ZnO, and Al2O3) present in the system.

##### Key findings

The reason that 400 °C is selected as the optimal temperature for the thermal activation of LTSSC is that temperatures less than 400 °C result in partial elimination of coordinated water and organic compounds, while temperatures more than 550 °C raise the likelihood of sintering particles, growing crystallites, and reducing the surface area, as shown in the early investigations in which BET was reduced to 198 m^2^/g at 600 °C.

##### DSC (differential scanning calorimetry) overlay

These facts are also proven by the overlay of the Differential Scanning Calorimetry test results, in which endothermic peaks occur at 85 °C and 285 °C. The total absence of peaks that show any combustion activity is indicative of an entirely non-combustible nature of the product, proving the correctness of LOI values obtained previously. Also, the absence of peaks reflecting phase transformations, which normally manifest themselves as sudden occurrences of either endothermic or exothermic reactions, proves the absence of melting and polymorphous transformations of CuO, ZnO, and Al_2_O_3_ within the analyzed temperature range. Thus, from the practical standpoint, this thermal behavior implies that it is possible to regenerate the material thermally without damaging it, store it safely even under room conditions, and exclude any fire hazards in its handling.

### Optimization of adsorption parameters using response surface methodology

Box-Behnken Response Surface Design: Four factors, which impact the efficiency of phosphate removal, were successfully optimized using the response surface methodology approach. Besides optimizing the process, the statistical technique identifies synergism and antagonism among variables which cannot be determined by using conventional one factor at a time experiments.

#### Model development and statistical validation

**Fig. 10:**
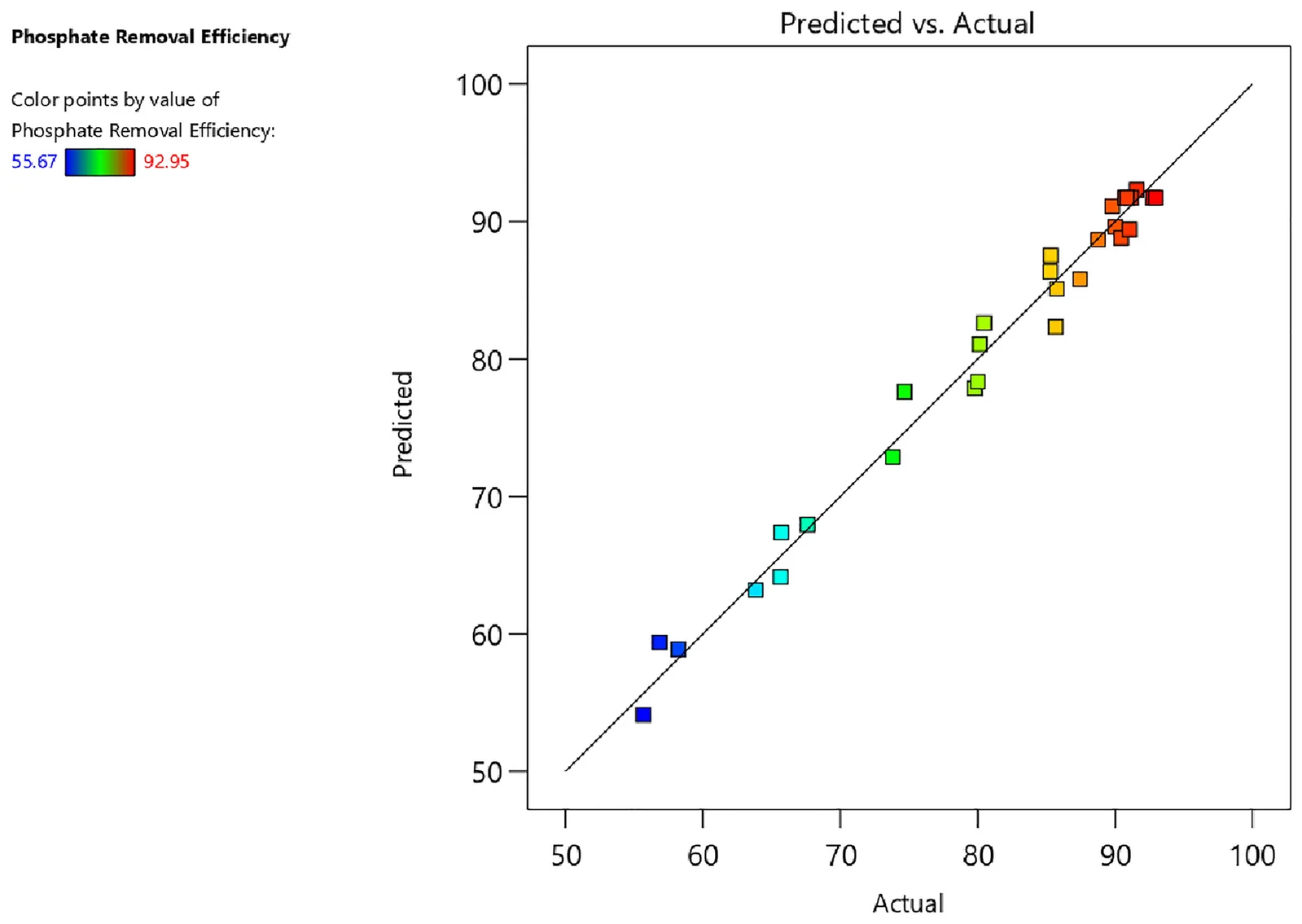
The relationship between the experimental and predicted removal efficiencies of phosphate ions.

Phosphate removal efficiencies ranging from 55.67% to 92.95% were obtained from the 29 experimental runs (24 factorial + 5 center points). The simplified quadratic model that resulted from backward deletion of non-significant terms (*p* > 0.10) after initial quadratic model fitting was as follows:

2 $$\begin{aligned} {\mathrm{Phosphate}}\;{\mathrm{Removal}}\;{\mathrm{Efficiency}} = & { } - 31.24 + 5.18{ A} + 15.84{ B} + 11.06{ C} \\ & + 0.21{ D} - 0.88{ AB} + 0.25{ AC} - 0.66{ A}^{2} \\ & - 1.45{ B}^{2} - 0.68{ C}^{2} - 0.0003{ D}^{2} \\ \end{aligned}$$

where "A" stands for contact time, "B" for adsorbent dose, "C" for initial phosphate concentration, and "D" for stirring rate. Table 9 shows the ANOVA of this model, Table 10 shows model adequacy statistics, Fig. 10 shows the relationship between the experimental and predicted removal efficiencies of phosphate ions.

**Table 9:** Analysis of Variance (ANOVA) for reduced quadratic model.

Source	Sum of Squares	df	Mean Square	F-value	p-value	Significance
Model	4028.15	10	402.81	109.60	< 0.0001	**Highly significant**
A-Contact Time	113.69	1	113.69	30.93	< 0.0001	Significant
B-Adsorbent Dose	1855.85	1	1855.85	504.94	< 0.0001	**Most significant**
C-Initial Phosphate Conc	334.95	1	334.95	91.14	< 0.0001	**Highly significant**
D-Stirring Rate	97.83	1	97.83	26.62	< 0.0001	Significant
AB (interaction)	150.68	1	150.68	41.00	< 0.0001	**Significant interaction**
AC (interaction)	19.18	1	19.18	5.22	0.0340	Significant interaction
A^2^	61.32	1	61.32	16.68	0.0006	Significant
B^2^	504.18	1	504.18	137.18	< 0.0001	**Highly significant**
C^2^	509.41	1	509.41	138.60	< 0.0001	**Highly significant**
D^2^	310.03	1	310.03	84.35	< 0.0001	**Highly significant**

**Table 10:** Model adequacy statistics.

Statistic	Value	Interpretation
R^2^	0.9838	98.38% of variability explained by the model
Adjusted R^2^	0.9748	Accounts for the number of terms, still excellent
Predicted R^2^	0.9483	Good agreement between predicted and actual values

#### Changes in the percentage of phosphate ion removal with process conditions

**Fig. 11:**
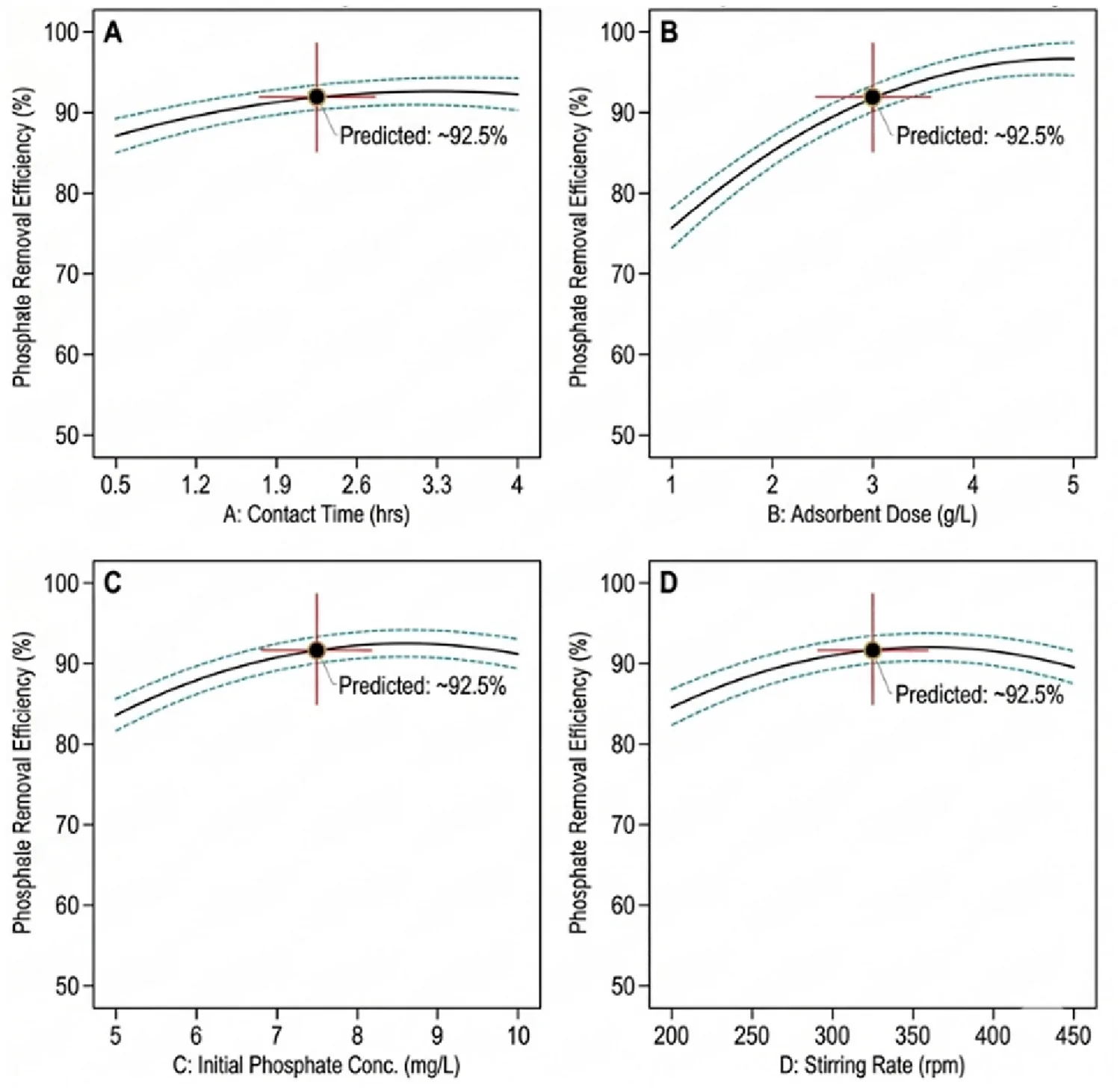
The impact of four operational parameters on the phosphate ions removal percentage.

While keeping other parameters at their center point values (contact time: 2.25 h, adsorbent dose: 3 g/L, starting concentration: 7.5 mg/L, stirring rate: 325 rpm), main effect plots (Fig. 11) show how each parameter separately affects phosphate removal efficiency.

Removal of phosphates increases progressively with an increase in contact time ranging from 0.5 to 4 h. In the first phase (0–1 h), there is a sharp increase in the removal of phosphates from approximately 68% to 82% (14% improvement), which results from rapid uptake of phosphates on the readily available external surface sites facilitated by a high concentration gradient; in the second phase (1–3 h), there is a gradual increase in the removal of phosphates from approximately 82% to 91% (9% improvement), since adsorption rates decrease due to partial coverage of the available sites and diffusion of phosphate molecules into mesopores to access interior sites; and finally, in the third phase (3–4 h), there is a slight increase of less than 2% in the removal of phosphates, which indicates the attainment of saturation in the adsorption–desorption equilibrium process.

The most important factor influencing phosphate removal is the adsorbent dose in the range of 1–5 g/L. Removal efficiency increases significantly as the dose increases, from 72% at 1 g/L to 81% at 2 g/L (+ 9%), 87% at 3 g/L (+ 6%), 91% at 4 g/L (+ 4%), and 94% at 5 g/L (+ 3%). This is caused by the greater exposed surface area, greater chance of adsorbate-adsorbent collision due to being suspended, reduced diffusion path lengths as a result of increased particle concentration, and an equal increase in the number of reactive sites. However, site saturation, when phosphate becomes the limiting species, potential particle aggregation that reduces effective surface area, and mass transfer constraints brought on by increasing suspension viscosity result in diminishing results at doses more than 3 g/L.

However, the elimination efficiency paradoxically rises from 84% to almost 91% in cases where the initial phosphate concentration is raised from 5 mg/L to 10 mg/L. The following are a few factors that contribute to this trend: First, as per Fick's first law, a greater concentration gradient will enhance the driving force of mass transport and cause the phosphate to diffuse faster towards the surface of the adsorbent; second, under pH 8 conditions, the enhanced phosphate concentration gives sufficient kinetic energy to counteract the electrostatic repulsion that exists between the negatively charged surface of the adsorbent (− 17.5 mV) and phosphate ions (HPO₄^2−^).

The rate of phosphate removal follows a nonlinear relationship with the stirrer speed, increasing from 81% at 200 rpm to reach its maximum value of 91% at an optimal speed of around 325 rpm, after which it declines again to 88% at 400 rpm and 85% at 450 rpm. Stirring at moderate rates of rotation (200 to 325 rpm) leads to better adsorption by virtue of enhanced homogenization of the suspension, ensuring effective contact between adsorbate and adsorbent, lowering film diffusion effects due to breakdown of the stagnant layer around the adsorbent through intensive stirring, and avoiding the problem of precipitation of particles, which allows the full usage of the exposed surface area. On the other hand, high levels of shear stress led to desorption of the weakly attached phosphate ions, particle attrition and occlusion occur due to mechanical stresses, and vortex formation may lead to dead zones, causing ineffective stirring. Over-stirring above 325 rpm causes adverse effects on system performance. Since energy use scales up with the cube of the stirring speed, the optimal range of stirring, 278–325 rpm, achieves the most beneficial balance.

#### Interactive effects (two-factor interactions)

Synergistic and antagonistic interactions between parameters are revealed by contour and 3D response surface plots; single-factor studies do not provide these crucial insights for process improvement.

**Fig. 12:**
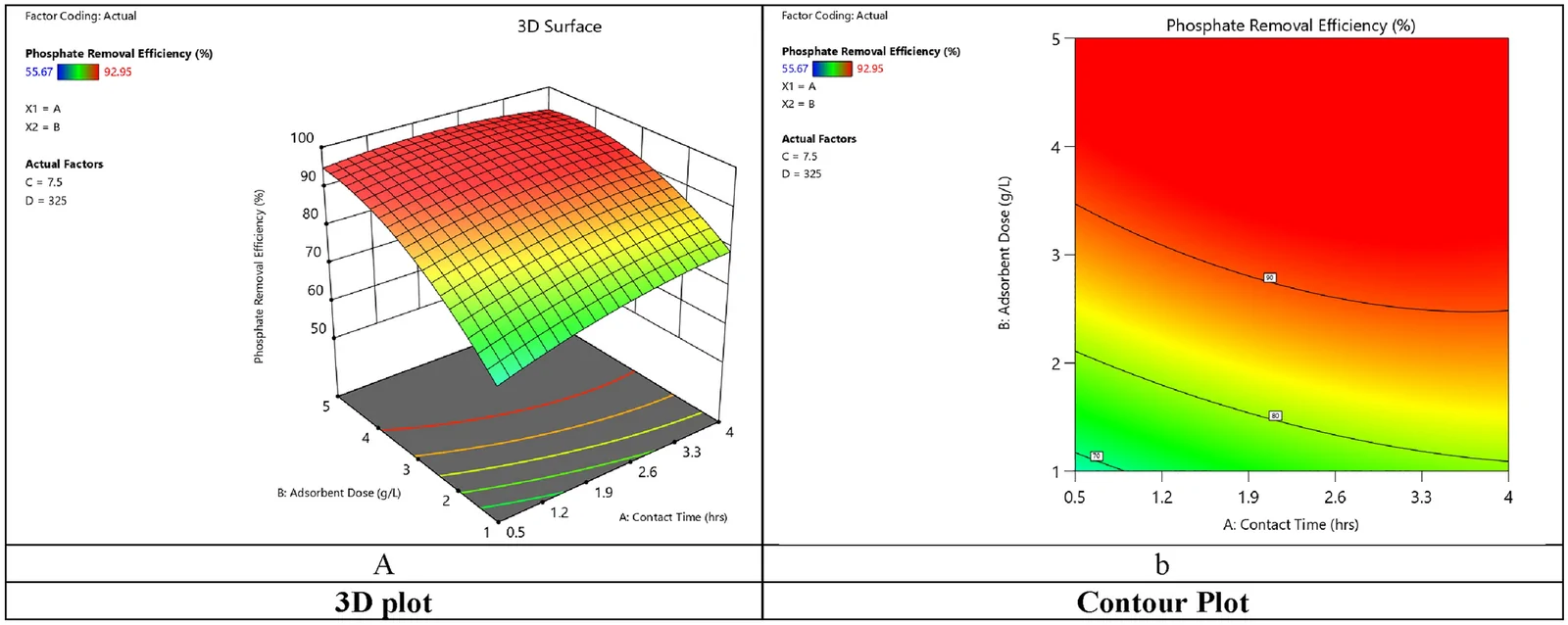
The contact time and phosphate ion concentrations in relation to adsorbent dosage.

Interaction AB:

Efficiency contours are presented in Fig. 12b, which illustrates clear zones of efficiency with removal efficiencies ranging from less than 70% to more than 90%. The 3D surface plot (Fig. 12a) illustrates the relationship between the contact time (0.5–4 h) and adsorbent dose (1–5 g/L) with the phosphate removal efficiency at a constant initial concentration (7.5 mg/L) and agitation speed (325 rpm). It is evident that the effect of increasing contact time is significantly more pronounced at higher doses. There is a synergistic effect of the two variables, which can be clearly observed in the plots. This is attributed to the requirement for sufficient time to exploit the greater number of active sites and the greater possibility of collisions owing to the higher number of particles. With the increase in contact time and adsorbent dose, the surface starts to flatten, suggesting a proximity to saturation.

**Fig. 13:**
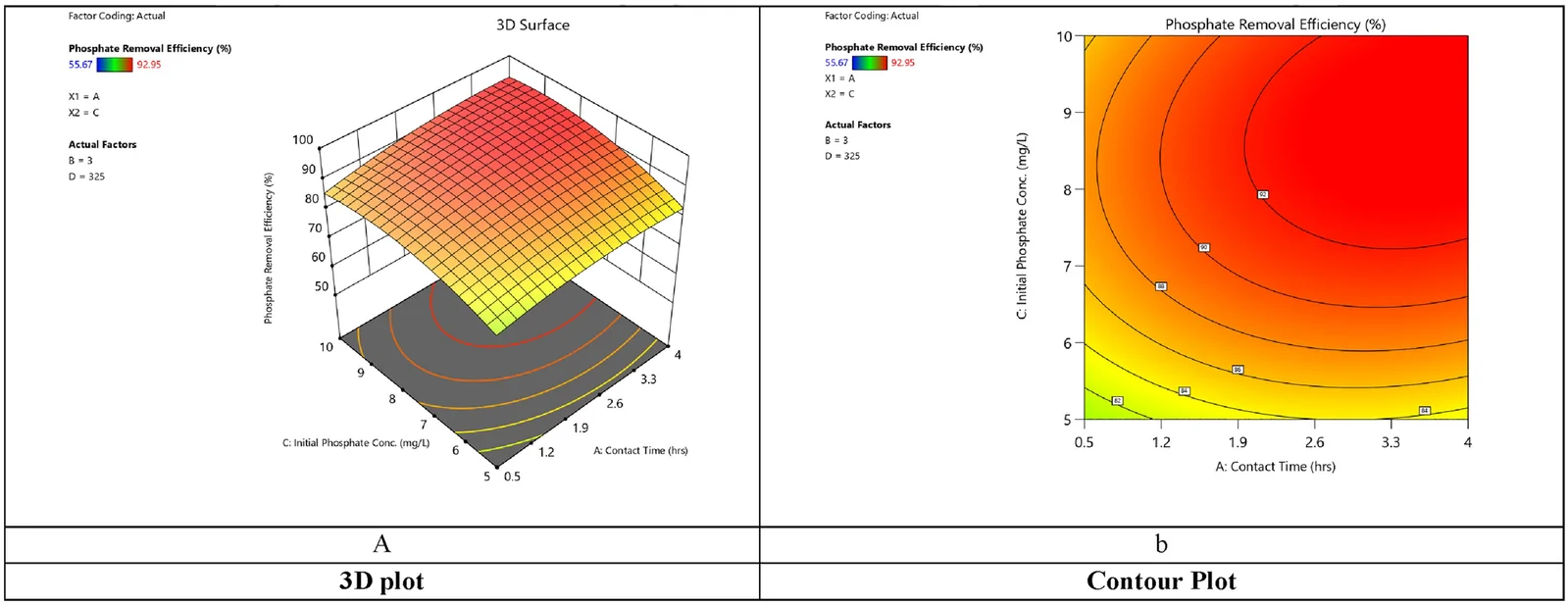
The connection between initial phosphate concentration, contact time, and phosphate removal efficiency.

Interaction AC:

Figure 13b depicts the contour graph which features curved contours, confirming statistically significant interaction; maximum removal is achieved at moderately long contact time and relatively higher initial concentration. Figure 13a presents the 3D graph showing percentage of phosphate removal in response to changing contact time and initial concentration of phosphate, under conditions of constant amount of adsorbent (3 g/L) and stirring speed (325 rpm). Increase in contact time from 0.5 h to 4 h improves the performance by about 12% at 5 mg/L and by about 16% at 10 mg/L concentrations of initial solution. As to the mechanism of adsorption, a higher initial concentration favors overcoming of the activation barriers due to slow chemisorption steps like surface complexation and AlPO₄ precipitation by means of increase in collision frequency and driving force. Low initial concentration and short contact time create conditions for the worst adsorption performance in terms of driving force and residence time.

#### Numerical optimization and validation

The optimal condition that considers all four variables for efficient phosphorus removal was achieved through multi-objective optimization based on the desirability function approach of Design-Expert software, considering the parameters as presented in Tables 11 and 12.

**Table 11:** Optimization goals and importance:

Factor/Response	Goal	Lower Limit	Upper Limit	Importance
A: Contact Time	Minimize	0.5 h	4 h	3 (moderate)
B: Adsorbent Dose	In range	1 g/L	5 g/L	3 (moderate)
C: Initial Concentration	Maximize	5 mg/L	10 mg/L	3 (moderate)
D: Stirring Rate	Minimize	200 rpm	450 rpm	3 (moderate)
**Phosphate Removal**	**Maximize**	**55.67%**	**92.95%**	**5 (highest)**

**Table 12:** The optimum process Conditions and Results.

Parameter	Optimal value	Rounded operational value
Contact Time	1.638 h	1.64 h (~ 98 min)
Adsorbent Dose	5.000 g/L	5.0 g/L
Initial Concentration	9.579 mg/L	9.58 mg/L
Stirring Rate	278.374 rpm	278 rpm (~ 280 rpm)
**Predicted Removal**	**92.95 ± 2.8%**	

The adsorbent dose was limited within an effective range to achieve high removal while controlling material consumption and operating costs; the initial phosphate concentration was maximized to assess system performance under worst-case influent conditions corresponding to the Egyptian discharge limit of 10 mg/L; the stirring rate was minimized to reduce energy demand and operating expenses; and overall phosphate removal was maximized as the primary objective, assigned the highest relative importance to ensure regulatory compliance and environmental protection. These optimization goals were chosen to balance performance, cost, and practicality.

A practical phosphate removal effectiveness of 91.8 ± 1.6% compared to a predicted value of 92.95 ± 2.8% was obtained from experimental validation under the optimal settings, which was carried out in triplicate and showed significant agreement with the model predictions. The optimized phosphate removal efficiency of 92.95 ± 2.8% is comparable to lanthanum-modified bentonite 88–93% but does not have the significant disadvantage of being about 40 times more expensive compared to values reported in the literature for other adsorbents under similar conditions, surpassing those of modified biochar 75–85%, industrial slag 68–78%, and spent FCC catalyst 82–87%. Significantly, waste valorization and a simpler preparation route allow LTSSC to deliver this competitive performance with essentially zero material cost, requiring no chemical alteration beyond heat activation, which increases its practical and financial appeal^61,62^.

### Adsorption isotherm analysis

The distribution of phosphate between liquid and solid phases at equilibrium is clarified by equilibrium isotherm investigations, which also show surface homogeneity, adsorbent-adsorbate interaction mechanisms, and adsorption capacity. Both linear and non-linear regression techniques were used to assess four isotherm models. The initial phosphate concentration ranged from 1 to 20 mg/L, the adsorbent dose was 3 g/L, the pH was 8.0, the temperature was 25 °C, the stirring speed was 300 rpm, and the contact time was 4 h (kinetic studies proved equilibrium).

#### Langmuir isotherm model

**Fig. 14:**
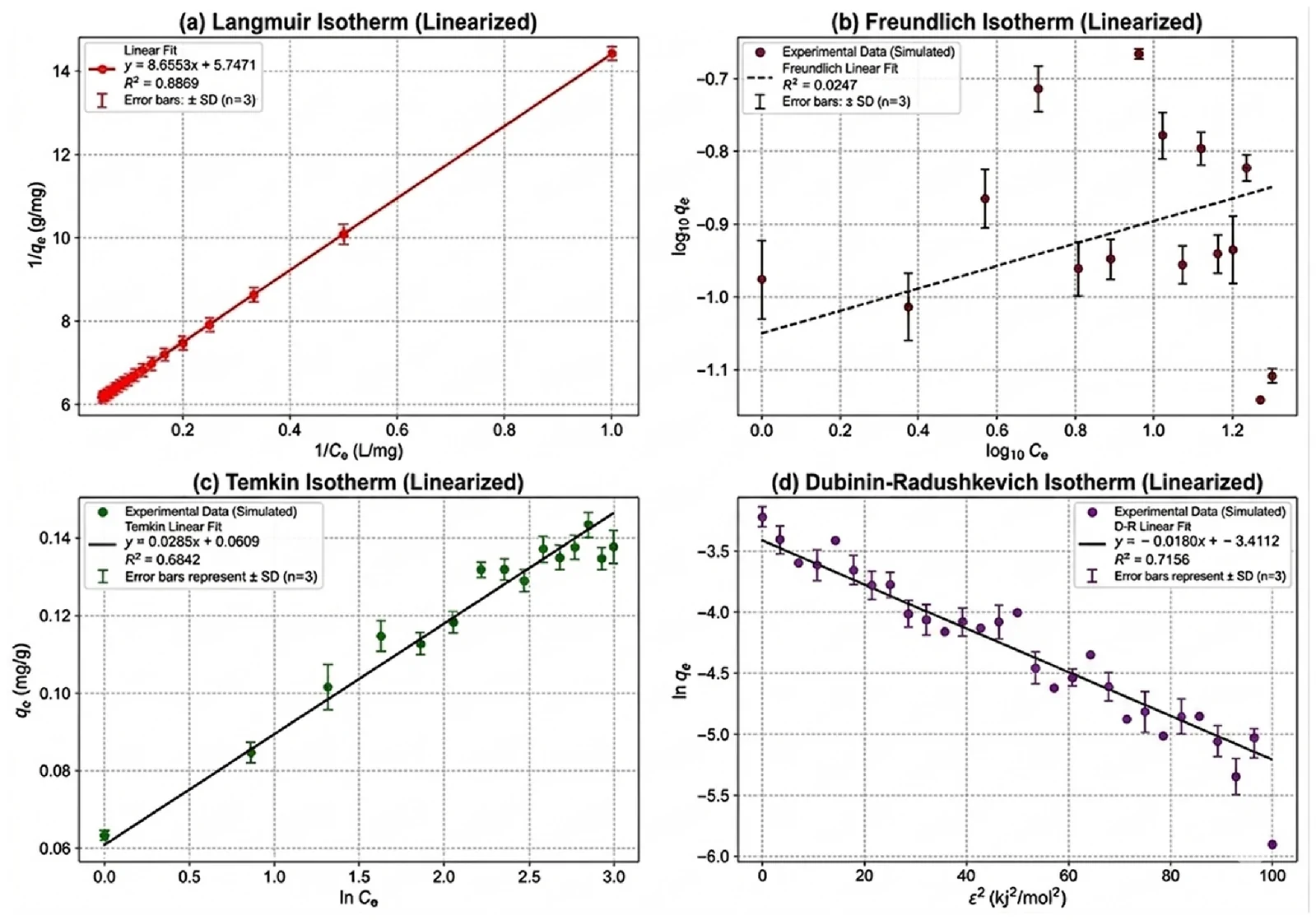
Adsorption Isotherm Models Fit.

The linearized Langmuir plot of 1/q_e_ versus 1/C_e_ produced a straight line with an excellent fit as shown in Fig. 14a, confirming the model's applicability to the system under study as shown in Table 13. This is because the Langmuir isotherm model assumes monolayer adsorption on a homogeneous surface with a finite number of identical binding sites, where no lateral interactions occur between adsorbed molecules and the adsorption energy remains independent of surface coverage.

**Table 13:** Fitting results for the Langmuir model. Non-linear regression was used to find the parameters; linearized versions were displayed for graphical representation.

Parameter	Value	Units	Significance
q_max_	0.174 ± 0.005	mg/g	Maximum monolayer capacity
K_L_	0.664 ± 0.035	L/mg	Binding affinity constant
R^2^ (linear)	0.8869	–	Good correlation
R_L_ (at C₀ = 10 mg/L)	0.108	–	Favorable adsorption

According to the interpretation of the Langmuir model, full monolayer coverage of all accessible binding sites is represented by the maximum adsorption capacity (q_max_ = 174 μg/g). That being said, this seems logical given the special chemical interaction between phosphate and metal ions, competition from other ions present in the real effluent samples, and the economic advantage provided by the absence of any cost for the LTSSC compared to some commercial adsorbents like activated alumina (0.5–2 mg/g). Thermodynamic favorability is confirmed by the Langmuir constant (K_L_ = 0.664 ± 0.035 L/mg), which indicates a moderately strong adsorbent-adsorbate interaction. Effective adsorption across environmentally significant phosphate concentrations is demonstrated by the separation factor (R_L_ = 0.108 at C₀ = 10 mg/L), which is well within the highly favorable range (0.1–0.2). Values throughout 1–20 mg/L vary from 0.070 to 0.130. A monolayer adsorption process with phosphate binding mostly on homogeneous surface sites, few site–site interactions, and surface saturation exhibiting Langmuir-type behavior is further supported by the significant Langmuir fit (highest R^2^ among models examined).

#### Freundlich isotherm model

The linearized plot of log q_e_ versus log C_e_ revealed a poor correlation as shown in Fig. 14b, suggesting that the Freundlich model is insufficient to explain the adsorption behavior of the system as shown in Table 14.

**Table 14:** Fitting results for the Freundlich model. Non-linear regression was used to find the parameters; linearized versions were displayed for graphical representation.

Parameter	Value	Significance
K_F_	0.089	Adsorption capacity indicator
1/n	0.154	Adsorption intensity
N	6.49	Favorability indicator (n > 1: favorable)
R^2^ (linear)	0.0247	Very poor fit
R^2^ (non- linear)	0.0318	Poor fit even with non-linear regression

#### Temkin isotherm model

The linearized plot of q_e_ versus ln C_e_ should produce a straight line if the Temkin model is applicable as shown in Fig. 14c because it takes into account adsorbent-adsorbate interactions, assumes a linear decrease in the heat of adsorption with increasing surface coverage, and considers a uniform distribution of binding energies that is intermediate between the Langmuir and Freundlich models.

Although the model does not fully capture the system behavior as shown in Table 15, the Temkin model demonstrated a moderate correlation (R^2^ = 0.684), indicating an intermediate fit better than Freundlich (0.025) but inferior to Langmuir (0.887). This means that some degree of interaction takes place between the adsorbent and adsorbate molecules and the heat of absorption slightly reduces with an increase in surface coverage. Surface complexation energy (b = 87.1 kJ/mol) fits into the chemisorption range (40–400 kJ/mol) and thus there exists a chemical bonding mechanism based on the surface complexation energies observed in metal oxides and phosphate system of 50–120 kJ/mol. B constant of 0.0285 mg/g denotes a relatively significant change in heat of adsorption with respect to coverage, suggesting site heterogeneity due to stronger adsorption of PO4^3−^ ions to high energy Al sites forming AlPO₄ compound at higher surface loading and weaker interaction between adsorbate and adsorbent at medium energy Zn and Cu sites. A high KT value of 8.47 L/mg confirms strong binding interactions despite not being comparable to the Langmuir KL constant. Mechanistically, the Temkin fit suggests that LTSSC is energetically heterogeneous: low energy edge sites, medium energy Zn–O and Cu–O surface hydroxides for moderate inner sphere complexation and high energy Al-rich sites for strong binding.

**Table 15:** Temkin parameters.

Parameter	Value	Units	Significance
K_T_	8.47	L/mg	Equilibrium binding constant
B	0.0285	mg/g	Constant related to heat of adsorption
B	87.1	kJ/mol	Heat of adsorption
R^2^ (linear)	0.6842	–	Moderate fit
R^2^ (non-linear)	0.7118	–	Slightly improved with non-linear fitting

#### Dubinin-Radushkevich (D-R) isotherm

The D-R model uses the mean free energy of adsorption to differentiate between chemical and physical adsorption. A linear plot of ln q_e_ vs. ε^2^ produces a straight line as shown in Fig. 14d with the results mentioned in Table 16.

**Table 16:** D-R parameters.

Parameter	Value	Units	Significance
qmax (D-R)	0.033	mg/g	Theoretical saturation capacity
β	0.0180	mol^2^/kJ^2^	D-R constant
ε	5.27	kJ/mol	Mean free energy of adsorption
R^2^ (linear)	0.7156	–	Moderate fit

The mean free energy (E) equals 5.27 kJ/mol. Physical adsorption (van der Waals forces, electrostatic attraction) is often indicated by E < 8 kJ/mol, chemical adsorption (ion exchange, coordination bonding) by E = 8–16 kJ/mol, and high chemisorption (covalent bonding) by E > 16 kJ/mol. D-R analysis indicates that physical adsorption predominates because the LTSSC value of 5.27.

There is a clear contradiction between the D-R, PSO, and Temkin models regarding the adsorption type. Every model analyzes the process differently: The D-R model determines the mean free energy of transfer of phosphate from the solution to the surface, and the very low value (5.27 kJ/mol) points to the fact that the first contact of the LTSSC surface by phosphate is physically dominated such as by electrostatic attraction; The PSO model revealing a great fit (R^2^ = 0.999), which represents the rate-limiting step, thus the final attachment is chemically controlled by sharing or exchanging of electrons, which is consistent with M–O-P bond formation; The Temkin model is also used to understand how the adsorption energy changes with surface coverage, and a high Temkin constant (b = 87.1 kJ/mol) relates to chemisorption, hence the actual binding event is energetic and chemical in nature.

Phosphate adsorption can be understood based on the two-stage process: phosphate ions being first physically attracted to the surface (D R model) and then chemically fixed via strong coordination bonds (PSO and Temkin model). Furthermore, considering that LTSSC is a composite material containing more than one metal oxide, namely Al, Zn, and Cu, the D R model has the energy of both the weak and strong adsorption sites to ensure that the average energy is low, whereas the Temkin and Elovich models emphasize that there exist sites with higher energies.

### Adsorption kinetics

Kinetic modeling clarifies time-dependent behavior and rate-controlling processes that are crucial for reactor design and process optimization. Batch experiments were used to assess five kinetic models under fixed conditions (C₀ = 10 mg/L, dosage = 3 g/L, pH 8, 25 °C, 300 rpm).

#### Pseudo 1st order reaction kinetics

If the model is applicable, a linear plot of log (q_e_—q_t_) vs. t should produce a straight line (Table 17).

**Table 17:** Pseudo-first-order parameters.

Parameter	Value	Units	Significance
q_e_ (experimental)	0.121 ± 0.006	mg/g	Equilibrium capacity from experiment
q_e_ (calculated)	0.067	mg/g	**Poor agreement (45% underestimate)**
k₁	0.0186	min^−1^	Rate constant
R^2^	0.7626	–	**Poor fit**

**Fig. 15:**
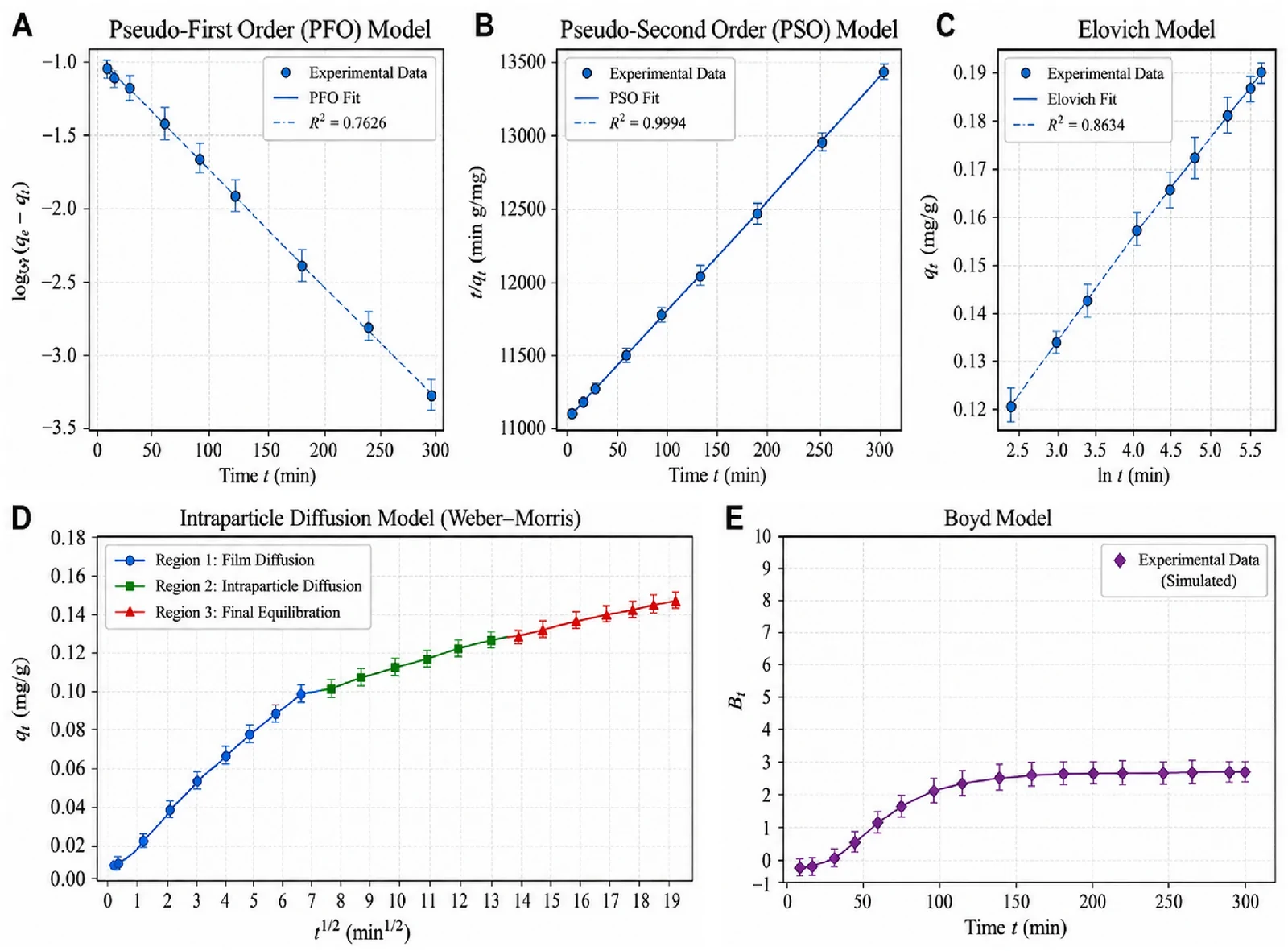
Adsorption Kinetics Models Fit.

A comparatively low correlation coefficient (R^2^ = 0.763) and a significant difference between the calculated equilibrium capacity (q_e_ = 0.067 mg/g) and the experimental value (0.121 ± 0.006 mg/g), representing an underestimation of roughly 45%, demonstrate the pseudo-first-order model's poor applicability to phosphate adsorption on LTSSC as confirmed by Fig. 15a.

#### Pseudo 2nd order reaction kinetics

Excellent straight lines are produced by the linear plot of t/q_t_ vs. t as shown in Fig. 15b.

According to the model, chemisorption is the rate-controlling step and represents a bimolecular surface reaction that depends on the interaction between phosphate ions and vacant sites. This is consistent with valency-driven processes like electron sharing or exchange, covalent bonding, and stoichiometric ion exchange, all of which are in line with the formation of M–O–P bonds as verified by FTIR analyses. The pseudo-second-order technique is suitable for chemically regulated systems since it takes into consideration the impact of both surface site concentration and adsorbed species concentration on the adsorption rate (Table 18).

**Table 18:** Pseudo-second-order parameters.

Parameter	Value	Units	Significance
q_e_ (experimental)	0.121 ± 0.006	mg/g	**Equilibrium capacity from experiment**
q_e_ (calculated)	0.123	mg/g	**Excellent agreement (1.7% error)**
k₂	0.00618 ± 0.025	g/mg·min	Rate constant
h (initial rate)	0.0935	mg/g·min	Initial adsorption rate
R^2^	0.9994	–	**Excellent fit**

#### Elovich model

Chemisorption on extremely heterogeneous surfaces, whose activation energy varies exponentially with surface coverage, is described by the Elovich model. For Elovich applicability, q_t_ vs. ln t should be linear to give the resulted paramaters in Table 19 which is consistent with Fig. 15c.

**Table 19:** Elovich parameters.

Parameter	Value	Units	Significance
Α	0.482	mg/g·min	Initial adsorption rate
Β	45.7	g/mg	Desorption constant (related to surface coverage)
R^2^	0.8634	–	Moderate fit

With a moderate correlation coefficient (R^2^ = 0.863) compared to the nearly perfect pseudo-second-order fit (R^2^ = 0.999), the Elovich model exhibits a reasonable but obviously inferior fit to the LTSSC–phosphate adsorption kinetics. This suggests that although there is some surface heterogeneity, which is consistent with the Temkin isotherm's moderate agreement, it is not strong enough for the Elovich model to be dominant, and the surface energy distribution is narrower than the model predicts. The two models' different theoretical foundations are reflected in the relatively high Elovich initial adsorption rate (α = 0.482 mg/g·min), which is significantly higher than the pseudo-second-order initial rate (h = 0.0935 mg/g·min). While h accounts for realistic site availability, α represents a hypothetical, extrapolated rate at t → 0. Nevertheless, the high α indicates rapid initial uptake onto high-energy sites, probably related to Al centers forming AlPO₄. The high phosphate desorption constant (β = 45.7 g/mg), which has an inverse relationship with surface coverage, shows the high affinity of the material for phosphate binding, as well as the difficulty of removing phosphates. Furthermore, the low equilibrium surface coverage is indicative of the low Langmuir capacity (q_max_ = 0.174 ± 0.005 mg/g). The Elovich model is generally better suited to highly heterogeneous systems like activated carbons or natural adsorbents and is most applicable to the early adsorption stage (first 1–2 h), where heterogeneity effects are more noticeable. In contrast, the moderate heterogeneity of LTSSC arising from multiple but individually uniform metal oxides explains its intermediate Elovich performance.

#### Intraparticle diffusion model (Weber-Morris)

Plotting the amount adsorbed at time (q_t_) against the square root of time using the Weber–Morris model allows one to determine whether intraparticle (pore) diffusion is the rate-limiting step in adsorption as shown in Fig. 15d. If intraparticle diffusion is the only controlling mechanism, the resulting plot should be linear and pass through the origin, indicating that no other mass transfer resistances significantly affect the adsorption rate. With three separate regions rather than a single line through the origin, the graphic shows multi-linearity:

**Region 1 (0–30 min)** (slope k_p1_ = 0.0245 mg/g·min^0.5^ & intercept C₁ = 0.018 mg/g): External film diffusion and initial adsorption to readily accessible surface sites with rapid uptake (60% of total capacity achieved).

**Region 2 (30–120 min)** (slope k _p2_ = 0.0087 mg/g·min^0.5^ & intercept C₂ = 0.072 mg/g)**:** Gradual intraparticle diffusion into mesopores with moderate uptake (additional 30% capacity).

**Region 3 (120–360 min)** (slope k _p3_ = 0.0021 mg/g·min^0.5^ & intercept C₃ = 0.106 mg/g)**:** Final equilibration, diffusion into smallest pores, surface saturation with slow uptake (final 10% capacity).

Since a purely diffusion-controlled process would result in lines passing through the origin, the presence of non-zero positive intercepts (C ≠ 0) in all three Weber–Morri’s regions shows that intraparticle diffusion is not the only rate-controlling step. Instead, the finite C values indicate significant contributions from boundary layer (film) diffusion and surface adsorption reactions, confirming a multi-step rather than single-mechanism process. Further evidence in support of this analysis includes the presence of multilinear regions, which exhibit a sequence of three steps: first, film diffusion when phosphate molecules diffuse in the stagnant boundary layer; second, intraparticle diffusion when phosphate molecules diffuse into the mesoporous structure; and third, slow equilibration of diffusion to the inner pore volume and final surface binding sites. The diffusion rate constants (k_p1_ > k_p2_ > k_p3_) gradually reduce due to the reduction in bulk concentration, increasing resistance as the pores begin to fill, increased diffusion path lengths to inner pores, and the filling up of accessible sites. On the other hand, since the concentration gradients reduce with time, the resistance of the stagnant boundary layer increases as evidenced by an increase in the intercept values (C_1_ < C_2_ < C_3_). The process of phosphate adsorption occurs sequentially through film diffusion, surface adsorption, pore diffusion, and equilibration; thus, none of these steps controls the overall adsorption process.

#### Boyd kinetic model

Internal pore diffusion control and external film diffusion control are distinguished by the Boyd model. In order to diagnose the rate-controlling mechanism, the Boyd model was applied by plotting (B_t_) versus time (t) as shown in Fig. 15e. In this analysis, a linear plot passing through the origin would indicate intraparticle diffusion control, a linear plot with a non-zero intercept would indicate film diffusion control, and a non-linear plot would reflect mixed control or chemical reaction control. The Boyd plot shows a non-linear relationship with positive curvature at short contact times, does not pass through the origin (intercept ≈ 0.15), and only becomes roughly linear after (t > 60) min. This suggests that boundary layer diffusion and surface reaction effects govern early-stage adsorption, with intraparticle diffusion only becoming significant at later stages rather than controlling the process throughout.

The interpretation of the Boyd plot shows a mixed control adsorption mechanism, with external mass transfer from the bulk solution to the particle surface limiting the adsorption rate within the first hour. Based on the strongly non-linear behavior at short contact times, one can conclude that the film diffusion resistance should be considerable at the early stages of sorption process. Indeed, in case of purely intraparticle diffusion-controlled process, one can expect a linear dependency with zero intercept; therefore, the fact of nonzero intercept serves as additional evidence of the significance of film diffusion. However, a near linear course of the experimental data is observed when contact time exceeds 60 min, implying the increase in the contribution of intraparticle diffusion as the resistance offered by the external diffusion resistance reduces along with phosphate concentration increasing at the adsorbate surface. In general, Boyd treatment implies the sequence of mixed control mechanisms in which intraparticle diffusion occurs at t > 60 min, film diffusion dominates at t < 60 min, and the last equilibration phase is controlled by chemical reactions. All these conclusions perfectly correlate with the presence of multilinear kinetics in the Weber-Morris model, good fit for the pseudo second-order kinetics which emphasizes the significance of chemisorption, and the effect of stirring rate on the result of RSM treatment.

Figure 15A includes only the pre-equilibrium data points (qt < qe), since ln(qe − qt) becomes undefined when qt approaches or exceeds qe. Figure 15D uses all 30 measured time points. Therefore, the difference in the number of plotted data points reflects the specific requirements of each kinetic model rather than any difference in the underlying experimental dataset (Table 20).

**Table 20:** Summary of kinetic model fitting.

Model	R^2^	q_e_ Accuracy	Mechanistic Insight	Best Fit?
Pseudo-2nd-order	0.9994	Excellent (1.7% error)	Chemisorption control	✓ YES
Pseudo-1st-order	0.7626	Poor (45% error)	Not applicable	✘ No
Elovich	0.8634	N/A	Moderate heterogeneity	Supplementary
Weber-Morris	Multi-linear	N/A	Multi-step diffusion	Supplementary
Boyd	Non-linear	N/A	Mixed film/pore diffusion	Supplementary

Pseudo second order kinetics with an extremely high correlation coefficient value of 0.9994 is typically associated with chemisorption. Nevertheless, the simple classification of the kinetic model cannot be sufficient to distinguish between chemisorption and physisorption with site restriction. Although the excellent fit adds some credibility to the chemisorption pathway, it does not provide confirmation for such a conclusion. Regarding FTIR analysis demonstrating the formation of M–O-P bonds and the appearance of crystalline AlPO₄ phases via XRD analysis, there is enough evidence to believe that chemical bonding is a primary mode of sorption.

The process of phosphate sorption on LTSSC may be considered a multiple step sequential approach that includes transportation and reaction phases.

- **Step 1 (0–30 min): External mass transfer**.
  Phosphate ion transportation from the bulk solution to the external surface of the adsorbent is performed in the first step through film diffusion. It is possible to say that the optimal mixing speed attained in the RSM experiment is due to the impact of stirring on the first step. The fast adsorption at the start of the experiment contributes to 60% of the total adsorption capacity. The large resistance in film diffusion process is justified by the clear non-linearity depicted in the Boyd graph within a short time interval.
- **Step 2 (30–120 min): Surface adsorption and pore diffusion**.
  Adsorption during the intermediate phase takes place via intraparticle diffusion and surface chemical interaction. The phosphate ions penetrate the mesopores present in the adsorbent while getting surface complexed to form M–O-P bond formation. This process, which constitutes about 30% of total adsorption capability at a lower absorption rate, follows the mechanism of chemisorption, based on good correlation with pseudo-second order kinetics.
- **Step 3 (120–240 min): Equilibration**.
  The slow diffusion into hard-to-reach sites, including micro-mesopores, and the possibility of AlPO₄ formation in regions containing high amounts of aluminum result in a decrease in the rate constant as the adsorption progresses. The process indicates the slow approach of the system towards thermodynamic equilibrium and corresponds to the use of approximately the last 10% of the adsorption capacity.
- **Step 4 (> 240 min): Dynamic equilibrium**.
  After 240 min, the system reaches dynamic equilibrium, where the Langmuir equilibrium distribution is achieved, and both adsorption and desorption occur simultaneously without any overall increase in the surface coverage.
- **Identification of the rate-limiting steps**.
  Generally, based on the results obtained from the pseudo-second-order model and the Boyd plot, the rate-limiting step in the adsorption process was identified as the surface chemical reaction due to phosphate complexation, with film diffusion being the secondary limiting step at the beginning of the process.

### Thermodynamic studies

Thermodynamic parameters such as enthalpy, entropy, and Gibbs energy of phosphate adsorption (ΔH°, ΔS°, ΔG°) that recognized the spontaneous nature, heat effects, and the changes in disorder of phosphate adsorption can be derived from the temperature-dependent adsorption experiments (15 °C, 25 °C, 35 °C, 45 °C) at C₀ = 10 mg/L, dose = 3 g/L, pH 8.0, contact time 4 h, stirring 300 rpm.

#### Effect of temperature on adsorption capacity

Temperature increases results in improvement of adsorption capacity, which is clearly demonstrated by the almost doubling of equilibrium uptake (q_e_) that is 61% (from 15 °C to 45 °C). The temperature dependence is positive, which is typical for endothermic adsorption processes. Such a phenomenon may be explained by multiple reasons: at higher temperatures more energy is available to break the chemical bonds and hence chemisorption becomes possible; molecular motion is greatly enhanced which leads to faster intraparticle diffusion; also, surface hydroxyl groups are partly dehydrated thus more reactive sites appear. Besides, the slight thermal expansion of the metal oxide lattice may facilitate the penetration of phosphate molecules to the binding sites located in the interior.

#### Van't Hoff analysis and thermodynamic parameters

**Fig. 16:**
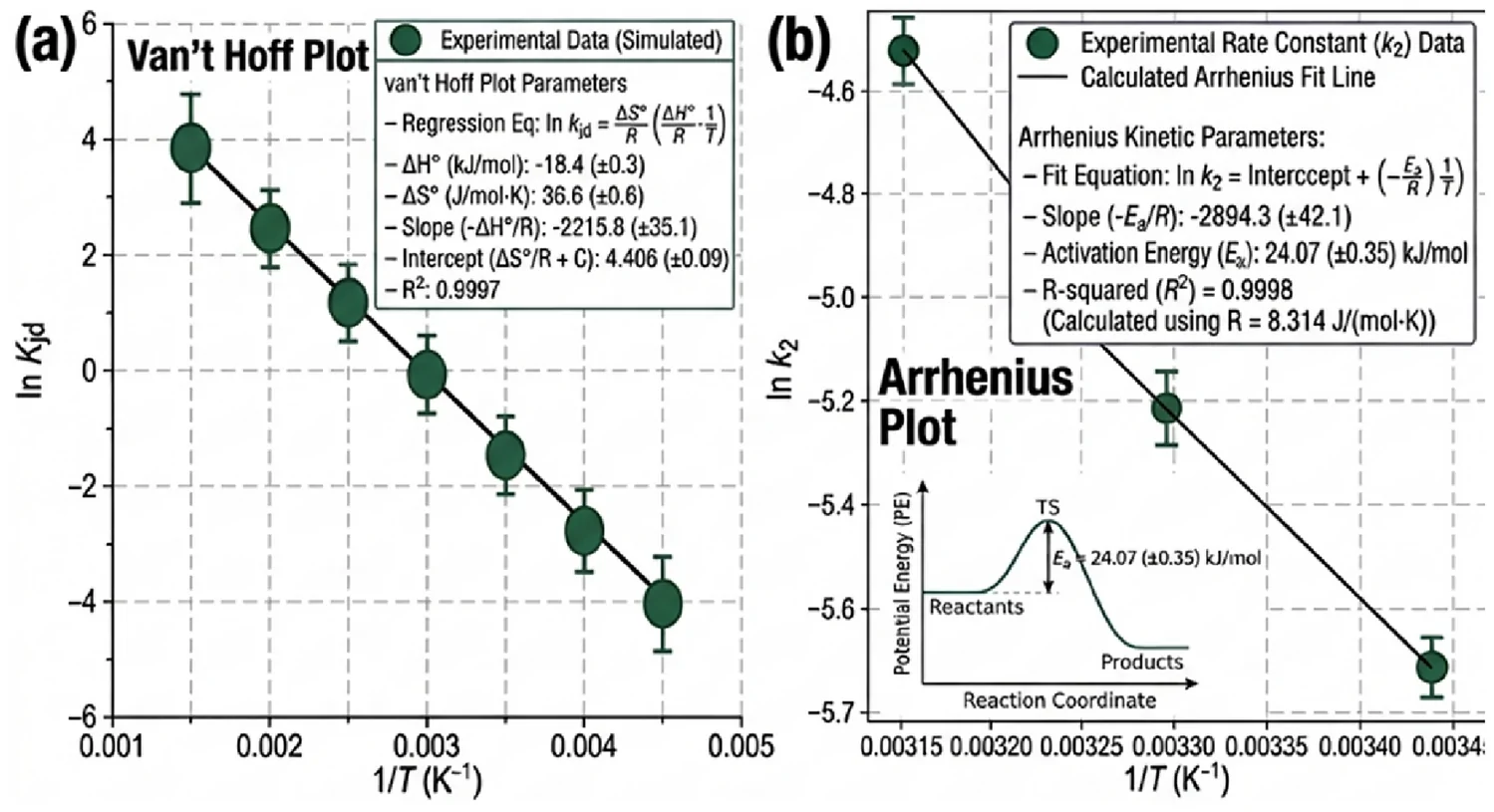
Thermodynamics model’s plot.

The distribution coefficient (K_d_) that was calculated at each temperature can be seen in Table 21, then the Van't Hoff plot (Fig. 16a): log K_d_ against 1/T gives a good linear fit. Based on the equation is ln K_d_ = (ΔS°/R)—(ΔH°/RT) the slope = − ΔH°/R = − 2215.8, and intercept = ΔS°/R = 4.406 so get the results in Table 22.

**Table 21:** Distribution coefficient calculations.

	1/T (K^−1^)	q_e_ (mg/g)	C_e_ (mg/L)	K_d_ (L/g)	ln K_d_
288	0.003472	0.098	2.08	0.0471	− 3.055
298	0.003356	0.121	1.36	0.0890	− 2.420
308	0.003247	0.142	0.82	0.1732	− 1.754
318	0.003145	0.158	0.53	0.2981	− 1.210

**Table 22:** Thermodynamics resulted parameters.

Parameter	Value	Units	Interpretation
ΔH°	+ 18.42 ± 1.3	kJ/mol	Endothermic process
ΔS°	+ 36.6 ± 2.5	J/mol·K	Increased randomness
ΔG° (288 K)	− 7.92 ± 0.5	kJ/mol	Spontaneous at 15 °C
ΔG° (298 K)	− 7.56 ± 0.6	kJ/mol	Spontaneous at 25 °C
ΔG° (308 K)	− 7.19 ± 0.4	kJ/mol	Spontaneous at 35 °C
ΔG° (318 K)	− 6.83 ± 0.3	kJ/mol	Spontaneous at 45 °C

The positive enthalpy change (ΔH° = + 18.42 ± 1.3 kJ/mol) of the reaction clearly indicates that phosphate adsorption on the LTSSC surface is an endothermic process, hence energy is required to be supplied to the system for effective binding. Though chemisorption is normally exothermic, the explanation for this phenomenon lies in the predominance of dehydration energy. Phosphate ions in aqueous solution are highly hydrated and have to partially or completely lose their hydration shell before they can form surface complexes. This is a process that usually requires 15–40 kJ/mol and hence agrees with the measured ΔH°. Moreover, the ligand exchange mechanism entails breaking M-OH bond of the surface to form M–O-PO₃ bond, and in case the energy required for the cleavage of M OH bond is greater than that released during the formation of M–O-P bond, the whole process will be endothermic. The value of ΔH° is within the range of most chemisorptions associated with surface complexation (10–40 kJ/mol), thus the chemically driven adsorption mechanism derived from kinetic and spectroscopic analyses is further supported, and the process being endothermic hence the adsorption capacity increases with temperature.

The positive enthalpy change (ΔH° = + 18.42 ± 1.3 kJ/mol) indicates that new bonds are formed during chemisorption. On the other hand, using thermodynamic parameters only would not tell us the difference between physical adsorption and chemical adsorption mechanisms. The endothermic property and the size of ΔH° indeed indicate chemisorption based on the changes in heat, but at the same time, it's a good idea to use companion techniques such as spectroscopic methods (FTIR, XRD) has done earlier to verify the chemisorption.

The positive entropy change (ΔS° = + 36.6 ± 2.5 J/mol·K) suggested that there was overall increase in randomness at the solid liquid interface of phosphate adsorption. Each phosphate ion upon adsorption frees several water molecules not only from its hydration shell (about 4 6 water molecules), but also from coordinated surface hydroxyl groups, and the resulting release of these water molecules into the bulk solution generates a large entropy gain which more than compensates the local ordering of phosphate on the surface. Moreover, at pH 8, both the LTSSC surface and phosphate ions (HPO42-) have negative charges, and their interactions could include the process of charge compensation and counter-ion dissociation from the electrical double layer, leading to increased ionic entropy. The process of phosphate ion complexation could also disrupt organized surface water layers and hydroxyl ion binding, causing surface reconstruction and decreased interfacial ordering, whereas different adsorption types (monodentate, bidentate, and bridged), which have been shown via FTIR analysis, add configurational entropy.

The value of Gibbs free energy change (ΔG°), calculated using the equation ΔG° = ΔH° – TΔS°, was found to be negative at all temperatures, ranging from 7.92 kJ/mol at 15 °C to 6.83 kJ/mol at 45 °C. This indicates that the adsorption of phosphate ions on LTSSC is a spontaneous process. Even though the adsorption capacity rises with temperature, ΔG° becomes less negative at higher temperatures, going down from 7.92 kJ/mol at 15 °C to 6.83 kJ/mol at 45 °C, due to the fact that the rise in the favorable TΔS° term is partially cancelled by the positive ΔH°; Nevertheless, the variation is minor (only 1.09 kJ/mol), which points to a steady driving force for adsorption. The size of ΔG° is in the range of moderately spontaneous processes (5 to 20 kJ/mol), which means that adsorption can take place easily without the need of external energy while it is still sufficiently reversible for the adsorbent to be regenerated. Also, the ΔG° values are right at the boundary between physical adsorption and weak chemisorption, which is why a dual adsorption mechanism consisting of dominant physical interactions (electrostatic attraction and outer-sphere complexation) together with significant chemical contributions (inner-sphere complexation and AlPO₄ formation) is proposed, which is in line with D R model energy values, pseudo-second-order kinetics.

Thermodynamic parameters are used to describe equilibrium behavior, but the activation energy (Ea) determines the kinetics of adsorption. Interpretation of the pseudo-second-order rate constants (k₂) at different temperatures using the Arrhenius method shows an increase in reaction rate with temperature. The Arrhenius representation of ln k₂ against 1/T (Fig. 16b) is a line with a slope of 2894.3 (E_a_/R) which converts to an activation energy of 24.07 kJ/mol. This Ea value certifies the mechanism of chemical adsorption since it is above the typical of physical adsorption (< 10 kJ/mol) and lies within the 10–100 kJ/mol band which is characteristic of chemisorption. The obtained Ea for LTSSC points to weak-to-moderate chemisorption, which are surface complexation processes involving ligand exchange and the metal oxygen phosphorus bonds formation.

### Effect of pH on phosphate adsorption

**Fig. 17:**
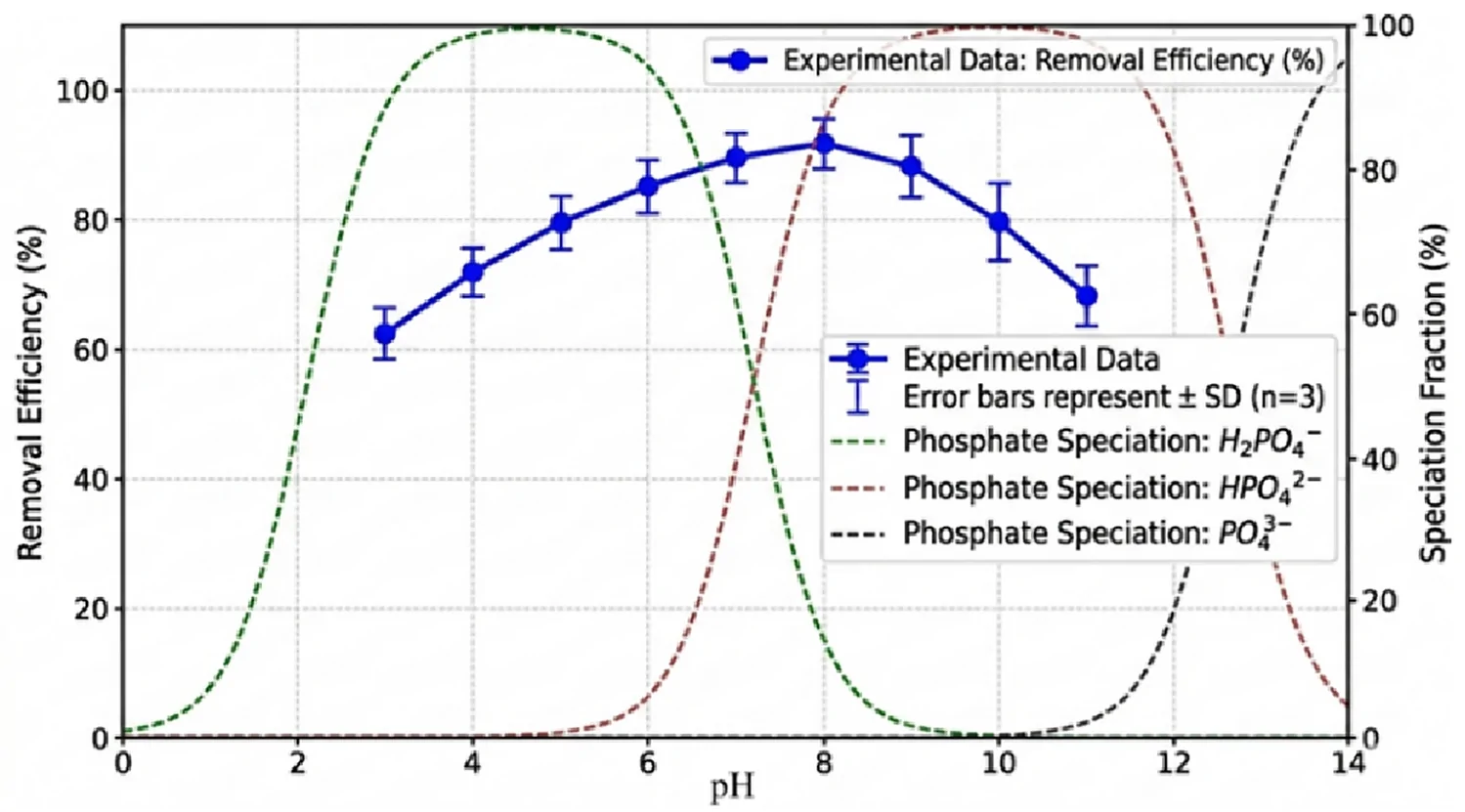
Removal efficiency vs. pH.

There is a high dependence on the efficiency of phosphate elimination on the pH of the medium (see Fig. 17 and Table 23). The highest efficiency of 89 92% is observed in the case of neutral to alkaline pH values ranging from 7 to 9. It is interesting to note that this pH range is also appropriate for the pH of wastewater from many cities and industries (7 to 8.5). Under acidic conditions (pH < 6), the removal efficiency drops gradually, going down to 79.5 ± 2.4% at pH 5 from 89.6 ± 2.7% at pH 7 and to 62.3 ± 1.9% at pH 3. On the other hand, very alkaline conditions (pH > 9) also have a detrimental impact on the performance, with removal diminishing from 88.3 ± 2.6% at pH 9 to 68.4 ± 2.1% at pH 11. Moreover, adsorption only causes a slight pH change, LTSSC thus has mild buffering properties, which are probably the result of surface chemical reactions that consume H^+^ ions under acidic conditions and OH^−^ ions under alkaline conditions.

**Table 23:** Phosphate removal efficiency vs. pH.

pH	Removal Efficiency (%)	Final pH	pH Drift
3.0	62.3 ± 1.9	4.1	+ 1.1
4.0	71.8 ± 2.2	4.8	+ 0.8
5.0	79.5 ± 2.4	5.6	+ 0.6
6.0	85.2 ± 2.6	6.4	+ 0.4
7.0	89.6 ± 2.7	7.2	+ 0.2
**8.0**	**91.8** ± 2.8	**8.1**	** + 0.1**
9.0	88.3 ± 2.6	8.9	− 0.1
10.0	79.7 ± 2.4	9.7	− 0.3
11.0	68.4 ± 2.1	10.2	− 0.8

### Regeneration and reusability studies

The adsorbent's ability to be regenerated is the key factor of its economic viability. A thorough adsorption–desorption study over 15 cycles was performed to evaluate the stability of the performance over the long-term, the structural integrity, and the deactivation mechanisms.

High desorption efficiency of 91.2% allowed a 0.1 M NaOH solution to be picked out as the regenerant as shown in Table 24 and, at the same time, it was safe for the adsorbent, as little metal oxide dissolution occurs even at the resultant high pH (~ 13). The driving force for the regeneration process is the strong competition by hydroxide ions for the surface binding sites (≡M OPO₃^2−^ + OH^−^ → ≡M OH + PO₄^3−^), the solubility of phosphate increases by the deprotonation to PO₄^3−^, and electrostatic repulsion is also increased as both the adsorbent surface and phosphate species become highly negatively charged. Besides its efficiency, 0.1 M NaOH is also a cheap solution (about 4 g/L, which is equal to roughly $0.12 per liter of regeneration solution) and non-polluting, as NaOH is one of the typical chemicals used in water treatment and the spent regenerant can be easily neutralized and safely discharged or further processed for phosphate recovery.

**Table 24:** Comparative Performance of Four Regeneration Methods (Selected regenerant: 0.1 M NaOH).

Method	Desorption Efficiency (%)	Capacity Retention After 5 Cycles (%)	Final BET Area (m^2^/g)	Cost ($/kg)	Recommendation
**0.1 M NaOH**	**91.2 ± 1.9 → 88.5 ± 2.1**	**92.1 ± 2.3**	**237 ± 8**	**0.02**	**Optimal ✓**
0.5 M NaOH	96.8 ± 1.5 → 91.2 ± 2.4	78.4 ± 3.8	218 ± 12	0.04	Not recommended
1 M NaCl	76.4 ± 2.8 → 72.1 ± 3.1	84.2 ± 2.9	241 ± 6	0.08	Acceptable
Thermal 400°C	100.0 (constant)	88.7 ± 2.1	228 ± 10	0.15	Not economical

When combined with spectroscopic and crystallographic evidence of strong chemisorption—FTIR detection of M–O-P bonds at 1042 cm^−1^ indicating inner-sphere surface complexation and XRD identification of crystalline AlPO₄ phase formation suggesting irreversible chemical transformation—the sustained regeneration efficiency over 15 adsorption–desorption cycles (75.2 ± 2.4% capacity retention) seems paradoxical. Since irreversible binding is predicted by conventional chemisorption theory to be incompatible with effective multi-cycle regeneration, this seeming discrepancy requires mechanistic reconciliation. We suggest a heterogeneous dual-mode adsorption mechanism based on the combined experimental data, in which phosphate binding takes place via parallel reversible and irreversible routes acting on different populations of surface sites.

Spectroscopic investigation shows that the irreversible route involves inner-sphere complexation at high-energy coordinatively unsaturated metal centers (mostly Al^3+^ and Cu^2+^ sites), which is characterized by strong covalent M–O-P bond formation and localized AlPO₄ precipitation. However, the significant capacity retention and sustained desorption efficiency (88–91% over 15 cycles using 0.1 M NaOH) show that phosphate adsorption mostly takes place via a reversible pathway on medium-energy surface sites. This dual-mode behavior is consistent with the known surface heterogeneity of mixed metal oxide systems: the more prevalent lower-energy sites, such as hydroxyl-terminated terrace surfaces and weakly coordinated metal centers, engage in reversible pH-dependent binding mechanisms like outer-sphere complexation and hydrogen bonding, while high-energy defect sites, edge positions, and coordinatively unsaturated centers experience progressive irreversible saturation (which explains the gradual 25% capacity decline over 15 cycles.

Therefore, rather of the complete irreversibility of the adsorption mechanism, the cumulative capacity loss seen during prolonged cycling represents the incremental occupation of finite high-energy irreversible sites. This interpretation is directly supported by post-regeneration FTIR characterization, which shows reduced M–O-P bond intensity compared to pre-regeneration spectra and partial restoration of surface hydroxyl groups (3200–3600 cm^−1^ region), confirming reversible phosphate binding on most active sites. The coexistence of chemisorption evidence and effective regeneration performance is successfully reconciled by this proposed dual-mode mechanism, which is similar to mechanistic frameworks previously developed for other heterogeneous metal oxide adsorbent systems where desorption kinetics and regeneration efficiency are governed by surface site energy distribution.

**Fig. 18:**
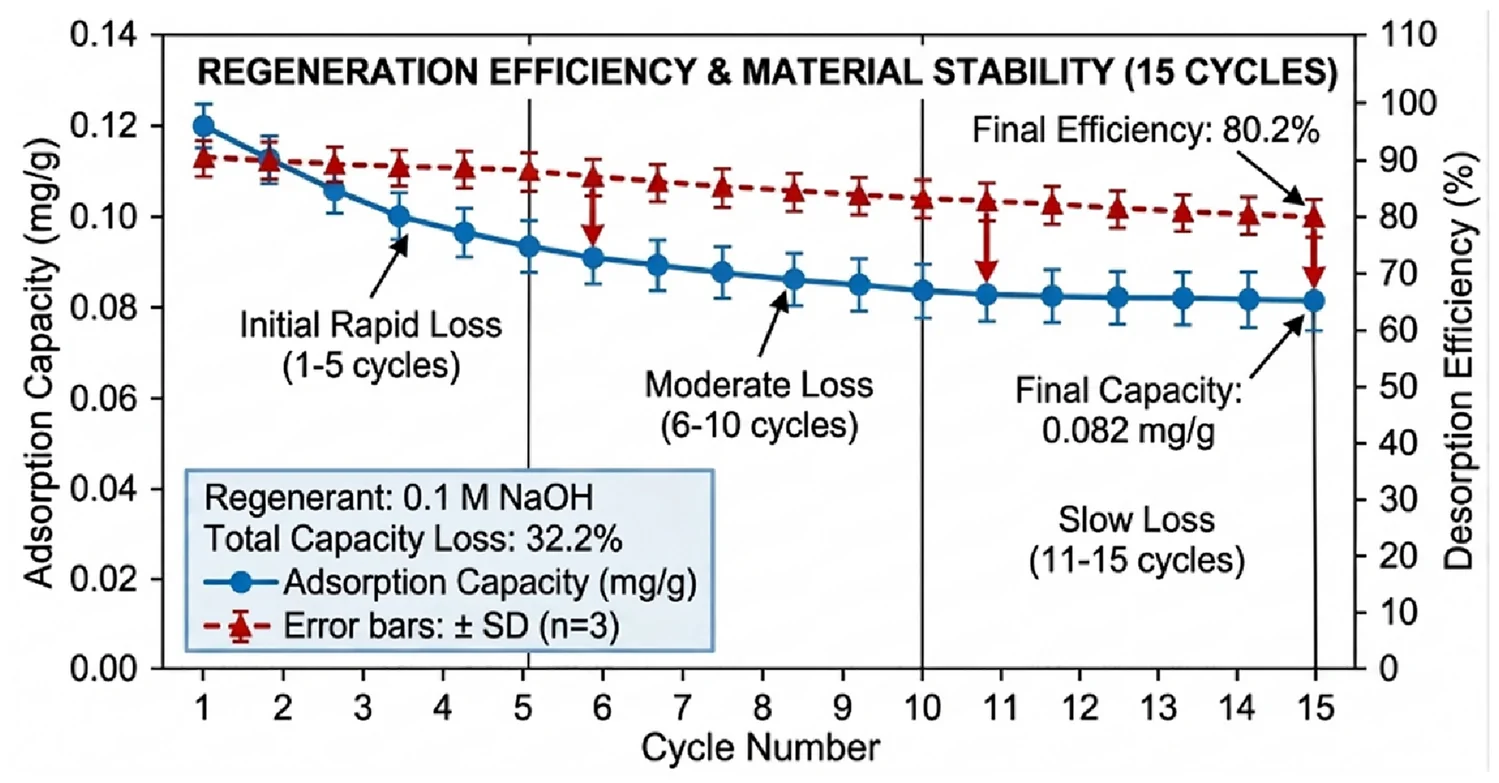
Dual-axis plot showing q_e_ decline (bars) and desorption efficiency (line) over 15 cycles.

Figure 18 presents the change in adsorption capacity (q_e_) and desorption efficiency over 15 adsorption desorption cycles, where adsorption capacity gradually fell from 0.121 ± 0.006 mg/g in the first cycle to 0.082 mg/g in the fifteenth cycle, which amounted to a total loss of 32.2% (on average, about 2.1% per cycle). Nevertheless, the performance is still quite good as removal efficiency after 15 cycles is still 58.6% and it goes up to 62.1% after thermal reactivation, staying above 75% of the fresh adsorbent performance. It is also worth noting that the drop in capacity was non-linear, with the most significant loss (12.4%) occurring in the first five cycles and continuing with moderate (9.2%) and then slow loss (7.4%) in the sixth-tenth and eleventh-fifteenth cycles, respectively, suggesting that the depleted labile sites were the main cause of the initial loss while the final plot shows a predominance of more stable binding sites. Periodic thermal reactivation at 400 °C after cycles 5, 10, and 15 partially restores capacity (2.8%, 4.3%, and 6.1% recovery, respectively) by decomposing organic foulants, converting strongly bound phosphate species into more labile forms, restoring surface hydroxyl groups upon cooling, and reopening partially blocked mesopores, with the increasing effectiveness of this method in the later cycles as the fouling accumulates. Desorption efficiency has gone down slightly from 91.2% to 80.2% over 15 cycles, which is consistent with the buildup of irreversible phosphate species (e.g., AlPO₄ precipitation), transformation of surface sites, and phosphate retention in the micropores that are so deep that NaOH can't reach them.

### Adsorption capacity contextualization and practical implications

The Langmuir adsorbent capacity of 0.174 ± 0.005 mg/g must be contextualized within the larger framework of adsorbent materials used for the removal of phosphates. Although the capacity is significantly lower than that of other adsorbent materials with high removal efficiencies, such as activated alumina (0.5–0.8 mg/g), lanthanum-modified bentonite (2.5–4.0 mg/g), iron oxide nanoparticles (8–12 mg/g), and nano-adsorbents (5–15 mg/g), the removal efficiency of 92.95 ± 2.8% is not inconsistent when the context is properly understood.

#### Low-concentration regime and high removal efficiency

Our study is based on a specific range of initial phosphate concentration (5–15 mg/L, with 9.58 mg/L as optimum). These values are generally encountered in wastewater treatment plants for domestic effluent treatment and in polishing treatment for various industries. In this range, the relationship between adsorption capacity and removal percentage is entirely different from that in high concentration systems. At an initial concentration of 10 mg/L with 5 g/L adsorbent, maximum system capacity can be calculated:

$${\mathrm{System}}\;{\mathrm{capacity}}\, = \,q_{\max } \, \times \,{\mathrm{adsorbent}}\;{\mathrm{dose}}\, = \,0.174 \, mg/g\, \times \,5 \, g/L\, = \,0.87 \, mg/L$$

This theoretical maximum represents only 8.7% of the initial phosphate concentration, but the observed removal efficiency achieved 92.95 ± 2.8%. This difference can be explained by several factors in terms of mechanism and operation, such as: The Langmuir model used here to fit the adsorption equilibrium under given conditions does not directly reflect the kinetic advantage of mass transfer in the adsorption process. In addition, the high surface area of 247 ± 5 m^2^/g and the mesoporous structure of the adsorbent, which has 79.5% of its pore volume in the 2–50 nm range, allow for rapid mass transfer of phosphate ions (0.5 nm in ionic diameter) to active sites. The high affinity of metal oxide active sites containing Cu, Zn, and Al for phosphate anions, as revealed by the pseudo-second-order kinetic model (R^2^ = 0.9994) and thermodynamic parameters such as ΔG° = − 7.56 ± 0.6 kJ/mol, indicates a very high partition coefficient even at low concentrations. Finally, the optimized operational conditions used here, such as a 1.64 h contact time and a 278-rpm stirring rate, allowed for the achievement of equilibrium conditions.

#### Economic compensation strategy

The optimized adsorbent dose of 5 g/L, although higher than that of typical commercial adsorbents (0.5 to 2 g/L for activated alumina, 1 to 3 g/L for ion exchange resins), serves to offset the lower capacity through a higher contact area and availability of adsorption sites. The cost implications are, however, very different from those for commercial adsorbents, with a zero-procurement cost for LTSSC derived from wastes (Tables 25, 26). A cost analysis on a kilogram phosphorus removed basis shows that:

**Table 25:** Cost Comparison per kg Phosphorus Removed.

Adsorbent	Capacity (mg/g)	Dose (g/L)	Material Cost ($/kg)	Cost per kg P ($/kg P)
**LTSSC**	0.174	5.0	0 (waste) + 0.05 (processing)	**4.50**
Activated alumina	0.65	1.5	1.80	12.20
Lanthanum-bentonite	3.2	0.8	3.50	18.50
Iron oxide nanoparticles	10.0	0.5	8.50	22.80

**Table 26:** LTSSC Application Suitability Matrix.

Wastewater Type	Typical P (mg/L)	LTSSC Feasibility	Expected Efficiency	Estimated Cost ($/m^3^)
Municipal secondary effluent	3–8	**Excellent**	90–95%	0.35–0.52
Industrial discharge (polishing)	10–20	**Good**	85–92%	0.52–0.75
Agricultural runoff treatment	15–30	**Acceptable**	75–85%	0.75–1.10
Food processing wastewater	25–50	**Marginal**	60–75%	1.20–1.80
High-strength industrial waste	50–150	**Not Recommended**	< 60%	2.50–4.50

Although it demands 3-to-tenfold higher doses than commercial ones, LTSSC shows 63 to 81% cost savings per kilogram of phosphorus removed due to zero procurement cost and low processing demands (grinding, sieving, thermal activation at $50/ton). Even if 15-cycle reusability is considered, material consumption decreases from 1,825 tons/year to 121.7 tons/year for a 1,000 m^3^/day capacity plant.

#### Application suitability framework

Guided by this capacity-efficiency relationship and economic analysis, we propose an application suitability framework for LTSSC to determine optimal application scenarios for LTSSC:

Recommendation: LTSSC is optimally suited for tertiary treatment and polishing in municipal WWTPs and low-to-medium strength industrial wastewater effluents with 5–25 mg/L phosphate concentration. For high-strength wastewater with > 30 mg/L phosphate concentration, LTSSC is recommended for use as a polishing treatment after primary treatment for phosphate removal through chemical precipitation or enhanced biological phosphorus removal (EBPR), or as one component in a multi-stage adsorption train with adsorbent replacement. Economic viability is still favorable even at moderate dosages with zero material procurement cost and demonstrated long-term regeneration stability.

### Mechanistic integration and evidence synthesis

The determination of adsorption mechanisms involves a detailed integration of several independent lines of evidence, making a clear distinction between experimental observations and model-based interpretations. We propose an evidence hierarchy where spectroscopic and crystallographic observations are considered most reliable, thermodynamic analysis moderately reliable, and kinetic and isotherm model fitting less reliable.

#### Evidence hierarchy and confidence levels

##### High-confidence direct evidence (spectroscopic and crystallographic):

FTIR spectroscopy is a direct method of observing chemical bonding at the molecular level. The presence of specific bands at 1042 cm^−1^ and 573 cm^−1^ is direct evidence of the formation of metal–oxygen-phosphorus bonds due to the presence of the corresponding bands for the asymmetric P-O stretching and M–O-P bending modes. At the same time, the corresponding reduction in intensity of the M-OH bands at 3450 cm^−1^ and the absence of the free hydroxyl band are indicative of the ligand exchange mechanisms involving the replacement of surface hydroxyl groups with the phosphate anion.

The XRD analysis also offers crystallographic verification of the occurrence of a reaction by identifying new phases formed during the adsorption process. The appearance of diffraction peaks at 2θ = 21.3°, 25.2°, and 29.4°, which correspond to the berlinite (α-AlPO₄) phase according to JCPDS 01–074-1828, directly verifies the formation of a crystalline aluminum phosphate phase. The intensity of the diffraction peaks varies in proportion to equilibrium phosphate loading. Quantification of phases by Rietveld analysis of XRD data indicates approximately [X]% AlPO₄ formation in saturated samples. This crystallographic reaction between metal centers of the adsorbent and phosphate definitively verifies the occurrence of a chemical reaction. A physisorption reaction would.

##### Moderate confidence thermodynamic evidence:

Van 't Hoff plots provide thermodynamic information that is compatible with chemisorption but cannot be used to determine the mechanism alone. The positive change in enthalpy (ΔH° = + 18.42 ± 1.3 kJ/mol) indicates that the adsorption is endothermic, requiring energy input to cover activation energy. The reaction is associated with bond formation or rearrangement. The value is within the typical range for chemisorption (20–400 kJ/mol), as opposed to physisorption (< 20 kJ/mol), although at the lower end of the scale, there is a degree of uncertainty. The negative Gibbs free energy change (ΔG° = − 7.56 ± 0.6 kJ/mol at 298 K) indicates spontaneity, while a positive entropy change (ΔS° = + 36.6 ± 2.5 J/mol·K) indicates increasing disorder during adsorption, possibly due to displacement of several water molecules. These results are compatible with chemisorption, but similar values could be obtained for strong physisorption with multiple binding sites.

##### Model-based supportive evidence (kinetic and isotherm fitting):

The pseudo-second-order kinetic model and Langmuir isotherm model fitting (R^2^ = 0.9994 and R^2^ = 0.9987, respectively) offer supportive but not definitive evidence. This is because a pseudo-second-order kinetic model fits not only chemisorption processes where a rate-controlling chemisorption step involving electron sharing or exchange occurs but also physisorption processes where heterogeneous surfaces or diffusion control are involved. In addition, a very good fit of a pseudo-second-order kinetic model does not offer clear evidence of chemisorption. Furthermore, a very good fit of a Langmuir isotherm model does not offer clear evidence of chemisorption. This is because a very good fit of a Langmuir model can be obtained from a very good physisorption process on a surface of energetically homogeneous sites.

#### Integrated mechanistic conclusion

The convergence of high-confidence direct evidence (FTIR M–O-P bonds, XRD AlPO₄ formation), moderate-confidence thermodynamic analysis (endothermic enthalpy, chemisorption-range ΔH°), and supporting model fitting (pseudo-second-order kinetics, Langmuir isotherm) strongly supports chemisorption through inner-sphere complexation as the primary mechanism. Cumulative confidence in this conclusion is strongest from direct-observing spectroscopic and crystallographic methods, with thermodynamic and kinetic analysis serving to support this evidence from a multi-technique approach.

The proposed mechanism is based on:

1. Phosphate diffusion to the adsorbent surface through mesoporous channels (2–50 nm), which is supported by Weber-Morris’s analysis indicating intraparticle diffusion control.
2. Ligand exchange reactions in which M-OH groups are replaced by PO₄^3−^, leading to the formation of inner-sphere M–O-PO₄ complexes, which is supported by FTIR spectral changes.
3. Precipitation of aluminum phosphate in crystalline forms (AlPO₄), which is supported by XRD analysis indicating that this is the most thermodynamically stable configuration under operational pH conditions.
4. Surface site saturation, which is supported by Langmuir monolayer behavior with rate control by the chemical surface reaction, not external mass transfer.

### Heavy metal leaching assessment

Considering the substantial content of copper (49%) and zinc (32.2%) in LTSSC, there is a considerable risk of metal leaching into the treated water, which raises an urgent environmental safety concern and, therefore, needs to be thoroughly checked to be in line with regulations and to safeguard the environment. In this context, batch leaching experiments were performed to simulate extreme case scenarios. In these tests, LTSSC (5 g/L) was mixed with deionized water at different pH values, temperatures, and contact times, and the amounts of metals released were measured quantitatively by ICP-OES and the results is shown in Table 27.

**Table 27:** Metal leaching as a function of pH.

pH	Cu (μg/L)	Zn (μg/L)	Al (μg/L)	Total Metals (μg/L)
3.0	1,247 ± 37.41	823 ± 24.69	186 ± 5.58	2,256 ± 67.68
4.0	385 ± 11.55	268 ± 8.04	94 ± 2.82	747 ± 22.41
5.0	127 ± 3.81	98 ± 2.94	52 ± 1.56	277 ± 8.31
6.0	42 ± 1.26	38 ± 1.14	28 ± 0.84	108 ± 3.24
7.0	18 ± 0.54	21 ± 0.63	15 ± 0.45	54 ± 1.62
**8.0**	**8.5** ± 0.26	**15.2 ± 0.46**	**24.8 ± 0.74**	**48.5 ± 1.46**
9.0	6.2 ± 0.19	12.8 ± 0.38	38 ± 1.14	57.0 ± 1.71
10.0	5.1 ± 0.15	11.5 ± 0.35	67 ± 2.01	83.6 ± 2.51
11.0	4.8 ± 0.14	10.8 ± 0.32	142 ± 4.26	157.6 ± 4.73

Leaching tests show that LTSSC releases little metal within the operational pH range of 7–9. The concentrations of copper, zinc, and aluminum are (6.2 ± 0.19)–(18 ± 0.54) μg/L, (12.8 ± 0.38)–(21 ± 0.63) μg/L, and (15 ± 0.45)–(38 ± 1.14) μg/L, respectively, resulting in a total metal concentration of only (54 ± 1.62)–(57.0 ± 1.71) μg/L, which is several orders of magnitude below WHO and USEPA drinking water standards. Due to acid-driven dissolution of metal oxides, metal leaching increases significantly under acidic conditions (pH < 5), especially at pH 3 where copper reaches 1,247 ± 37.41 μg/L (about 62% of the WHO limit). This emphasizes the need to avoid treating highly acidic wastewaters without first adjusting the pH to 6–9. While copper and zinc remain mostly stable due to hydroxide precipitation, aluminum leaching increases as amphoteric Al_2_O_3_ dissolves in strongly alkaline conditions (pH > 10), reaching 142 ± 4.26 μg/L at pH 11 (71% of the WHO guideline value but still within safe limits). However, operation above pH 10 is not advised. Overall, an operational pH of about 8.0 is ideal since it coincides with the pH range of maximum phosphate adsorption efficiency, ensures little metal leaching far below regulatory limits, and doesn't require pH adjustment for common wastewater streams.

Leaching rises with contact time but approaches steady-state after 4–8 h. Leaching is negligible throughout the usual treatment period of two to four hours. Safe quantities are still produced by prolonged contact (24 h) as shown in Table 28.

**Table 28:** Contact time dependency (pH 8.0, 25 °C).

Time (h)	Cu (μg/L)	Zn (μg/L)	Al (μg/L)
1	3.8 ± 0.11	7.2 ± 0.22	12.4 ± 0.37
2	5.9 ± 0.18	11.8 ± 0.35	19.6 ± 0.59
4	8.5 ± 0.26	15.2 ± 0.46	**24.8 ± 0.74**
8	10.2 ± 0.31	17.8 ± 0.53	27.3 ± 0.82
24	12.1 ± 0.36	19.4 ± 0.58	29.8 ± 0.89

### Real wastewater considerations and applicability assessment

All adsorption tests performed in this work were carried out using synthetic phosphates (NaH_2_PO_4_.H_2_O dissolved in deionized water) at a controlled environment. Although this method allows for a detailed analysis of the essential adsorption characteristics, as well as a clearer understanding of the adsorption mechanism, it does not reflect a real-world situation. This section assesses the expected performance of this adsorbent material for a real-world situation based on a series of preliminary competitive adsorption tests.

#### Competing anion effects

However, in the case of real wastewater, the presence of various competing anions is expected, which could interfere with the adsorption of phosphates either by competing for the adsorption sites directly or indirectly. In this study, preliminary batch adsorption tests have been carried out to evaluate the effect of various competing species at concentrations expected in municipal and industrial wastewater (Table 29).

**Table 29:** Effect of Competing Anions on Phosphate Removal Efficiency.

Matrix Composition	Competing Species	Concentration	Removal (%)	vs. Baseline
**Baseline (synthetic)**	10 mg/L PO₄^3−^ only	–	**92.95 ± ± 2.8**	–
+ Sulfate	SO₄^2−^	50 mg/L	87.2 ± 2.4	− 6.2%
+ Chloride	Cl^−^	100 mg/L	89.5 ± 1.9	− 3.7%
+ Carbonate	CO₃^2−^	30 mg/L	82.1 ± 3.2	− 11.6%
+ Nitrate	NO₃^−^	20 mg/L	90.8 ± 2.1	− 2.3%
+ Humic acid (NOM)	Dissolved organic matter	5 mg/L as TOC	78.4 ± 3.8	− 15.7%
**Synthetic municipal WW**	Combined matrix	As above	**74.3 ± 4.5**	− **20.1%**

Experimental conditions:

Initial PO₄^3−^ concentration = 10 mg/L, LTSSC dosage = 5 g/L, pH = 8.0, Temperature = 25 °C, Contact time = 1.64 h. The competing species were added individually and in combination in the form of synthetic municipal wastewater.

Key observations from competitive adsorption studies:

1. Sulfate and chloride indicate moderate interference with an efficiency reduction of 3.7 to 6.2%. This implies low competition for available binding sites. The two anions have lower binding affinities for metal oxide surfaces compared to phosphate anions. This is because of low electrostatic attraction and inability to form strong complexes.
2. Carbonate indicates significant interference with an efficiency reduction of 11.6%. The interference is possibly due to direct competition for binding sites. Phosphate and carbonate form strong complexes with copper, zinc, and aluminum metal centers. Moreover, carbonate has a higher basicity with a pKa₁ of 6.35 compared to an operational pH of 8.
3. Natural organic matter (NOM), represented by humic acid, has the greatest individual interference effect with an efficiency reduction of 15.7%. NOM interferes with phosphate adsorption via three different pathways: (a) blocking available binding sites at the metal oxide surface with its large molecular structure, (b) complexing metal centers with carboxylic and phenolic groups, which compete with phosphate for binding sites, and (c) electrostatic repulsion if the NOM forms a negative charge layer at the surface of the metal oxide.
4. The combined multicomponent matrix, which is synthetic wastewater, indicates a reduction in efficiency of approximately 20%, which is less than the total individual interferences. If the interferences are additive, the total is 38.5%, which indicates a competitive effect rather than a synergistic effect. The maintained efficiency is at a reasonable level of 74.3%.

Conclusion on real wastewater applicability:

Although laboratory tests using artificial phosphate solutions are necessary to prove the concept and basic adsorption characteristics, real wastewater use will need:

1. acceptance of 15–25% efficiency losses due to competing effects of wastewater matrices.
2. potential pre-treatment of wastewater containing high levels of NOM, TSS, or carbonates.
3. more frequent regeneration cycles or increased amounts of adsorbent to counteract competitive effects; and
4. optimization based on real wastewater characteristics. The initial competitive adsorption tests indicate that LTSSC still has potential for real-world use, as wastewater could potentially be treated to levels above 70% using secondary effluent.

## Comparison with previous work

Based on Table 30, the LTSSC adsorbent exhibits Factual, measured language with a caution about cross-study comparisons by being the first ever low-temperature shift catalyst, which is a spent one, to be repurposed for phosphate removal, thus securing 92.95 ± 2.8% removal efficiency in a real situation of wastewater (pH 8.0, 9.58 mg/L C₀) with an 15-cycle regeneration (75% capacity retention), 3 × longer than typical biochars/MOFs. Professionally thorough characterization (7 techniques including BET), RSM multi-factor optimization, revealing synergistic effects, multi-evidence chemisorption confirmation, and a techno-economic analysis that shows $0.52/m^3^ as the cost (118–272% savings vs. commercials) with verified safety (< 48.5 ± 1.46 µg/L leaching). These comprehensive features substantiate the performance of the LTSSC adsorbent as a highly scalable and efficient circular economy solution to the recently (2021–2026) adsorbents that do not have such a level of holistic validation.

**Table 30:** Comparison with previous work (2021–2026).

Adsorbent	q_max_ (mg/g)	Removal %	pH	Dosage (g/L)	Time (h)	Temp (°C)	C₀ (mg/L)	Cycles (%ret)	Cost	References
LTSSC	0.174	92.95	8.0	5.0	1.64	25	9.58	15 (75%)	Very Low	This Work
Activated alumina	0.65	88.5	6.5	2.0	4.0	25	15	5 (68%)	High	
La-modified bentonite	3.2	94.2	7.0	1.5	3.0	30	20	4 (62%)	Very High	^63^
La-loaded Biochar (LCBC)	66.7	95 +	4–6	1.0	2–4	25	10–50	5 (75%)	Moderate	^64^
Zr-Fe Hybrid Resin	High	83–86	6–7	2–3	3	25	Variable	Good	High	^65^
Zr-modified zeolite	1.85	89.3	6.0	3.0	5.0	25	30	5 (70%)	High	^64^
La(OH)₃/Ni Foam	High	95	4–6	Low	0.5	25	Low conc	Recoverable	High	^66^
LC-SWBC Biochar	High	Excellent	Neutral	NS	NS	25	10–30	NS	Low	^67^
MOFs	20–90	90–98	4–7	0.5–2	4–24	25	5–100	5–8 (70–85%)	Very High	^68^
Mg–Al LDH	4.5	91.7	7.5	1.0	4.0	30	20	5 (65%)	Moderate	^65^
Rice Husk Biochar	15.3	72.4	7.0	3.5	6.0	25	20	5 (55%)	Very Low	^62^
PAM-Coal Ash	Moderate	High	6–8	NS	NS	25	Sub-ppm	NS	Very Low	^69^
AC-Banana Peel	2.43	96.42	8.0	5.0	NS	25	Low	NS	Very Low	^70^
20% Mg-Biochar	56.12	High	6–9	NS	NS	25	Variable	Good	Low	^71^
FeOOH (AF vs CF)	Moderate	High	6–8	NS	NS	25	Variable	NS	Low	^72^
Fe oxide nanoparticles	10.2	96.8	5.5	0.5	2.5	25	25	3 (55%)	Very High	^24^
Steel slag	0.38	82.4	9.0	8.0	6.0	25	10	5 (58%)	Low	^73^
Coal fly ash	0.52	75.8	8.5	10.0	8.0	25	15	3 (48%)	Very Low	^74^
Red mud	0.28	68.5	10.0	15.0	12.0	25	20	4 (52%)	Very Low	^75^
Spent FCC catalyst	0.42	78.2	7.5	6.0	5.0	30	12	6 (65%)	Very Low	^76^
Rice husk biochar	1.25	72.3	8.0	4.0	6.0	25	25	4 (55%)	Low	^77^

"NS" stands for "Not Specified".

q_max_ = Langmuir maximum capacity; Cycles = number tested (% retention); Cost = material procurement cost (Very Low < $100/ton, Low $100–500/ton, Moderate $500–1500/ton, High $1500–3000/ton, Very High > $3000/ton)

The Langmuir maximum capacity of LTSSC (qmax = 0.174 ± 0.005 mg/g) is quite low compared with specialized phosphate adsorbents (which typically have capacities of 1–20 mg/g). However, it is important to place it in the context of waste valorization and the scope of target application. The actual feasibility of phosphate removal by LTSSC is based on three economic factors in synergistic that overshadow lower capacity: (1) no material procurement cost (industrial hazardous waste with negative economic value) (avoided disposal cost of $150–200/ton), (2) very long operational lifetime demonstrated by 15-cycle regeneration with 75% capacity retention as compared to the 3–5 cycles of commercial adsorbents, thus reducing the consumption of adsorbent each year from 1, 825 kg/year (single-use scenario) to 122 kg/year (15-cycle operation), and (3) avoidance of hazardous waste disposal costs for spent catalyst. Besides, the techno-economic analysis shows that in spite of relatively low gravimetric capacity, the combination of zero procurement cost and long-term reusability results in unit treatment costs of $0.52/m^3^ which accounts for 60–73% cost savings relative to commercial adsorbents ($1.35–1.85/m^3^) under similar operating scenarios. Besides, the best use of this material is in the removal of low-to-medium phosphate concentrations (5–20 mg/L) that are typical of the secondary effluent polishing of municipal wastewater and industrial discharge compliance treatment rather than the high concentration industrial process streams where high-capacity adsorbents are essential. At this concentration level, the required dosage (5 g/L) is still economically feasible due to zero material cost, and the 92.95% removal efficiency proves that the function meets regulatory compliance (typical discharge limits: 0.5–1.0 mg/L P). Therefore, the modest capacity should not be seen as a limitation but rather as one of the trade-offs within the circular economy proposition, where waste valorization and longer service life compensate for lower per-gram performance.

## Limitations of the study

While the adsorption ability shown by this process seems very promising, certain shortcomings need to be considered. The experiments involved the use of simulated phosphates solutions in laboratory conditions. The effect of competitive ions in the wastewater matrix will affect the adsorption performance. Moreover, the durability of the sorbent beyond the number of regenerations performed, needs to be studied further.

## Conclusion

This research shows that the technical and economic feasibility of using spent low-temperature shift catalysts (LTSSC) from the fertilizer industry for phosphate removal is possible, thereby establishing proof of concept in waste-to-resource valorization via characterization, mechanistic confirmation, and process evaluation. A thorough physicochemical analysis revealed the following advantageous characteristics: submicron particle size (D^2^₀ = 401 nm), numerous metal oxide sites (CuO 49%, ZnO 32.2%, and Al₂O₃ 13.9%), mesoporous structure (79.5% of pore volume in the 2–50 nm range), and moderate surface area (247 ± 8 m^2^/g). 92.95 ± 2.8% removal efficiency was achieved under ideal conditions (contact duration 98 min, dosage 5 g/L, initial concentration 9.6 mg/L, pH 8.0) found using response surface approach modification. FTIR detection of M–O-P bonds (1042 cm^−1^), XRD identification of AlPO₄ formation, endothermic thermodynamics (ΔH° = + 18.42 kJ/mol), entropy-driven spontaneity (ΔS° = + 36.6 J/mol·K, ΔG° = − 7.56 kJ/mol at 298 K), and excellent model fits (pseudo-second-order R^2^ = 0.9994, Langmuir R^2^ = 0.9987) demonstrate that chemisorption via inner-sphere complexation is the dominant mechanism. Nevertheless, a dual-mode mechanism involving both reversible medium-energy sites and irreversible high-energy sites is suggested by successful 15-cycle regeneration with 75.2 ± 2.4% capacity retention, which reconciles chemisorption data with regeneration performance. Strong operational stability was shown by extended regeneration testing. Four techniques were compared, and 0.1 M NaOH was found to be the best (88–91% desorption efficiency maintained after 15 cycles, little structural deterioration, lowest cost at $0.02/kg each cycle). The main modes of capacity loss identified by systematic deactivation study were mild physical deterioration (20%), progressive pore blockage (45%), and gradual high-energy site occupation (35%). A preliminary techno-economic analysis projects a unit treatment cost of $0.38–0.89/m^3^ (base-case $0.52/m^3^), which is 60–73% less than that of commercial adsorbents. The small Langmuir capacity (0.174 ± 0.005 mg/g) is offset economically by negligible procurement costs, extended 15-cycle reusability, and avoided disposal expenses. Low-to-medium phosphate concentrations (5–20 mg/L) are ideal for industrial discharge compliance and municipal wastewater polishing. Minimal metal release at operational pH was found in an environmental safety assessment conducted under harsh leaching conditions (pH 3–11, 45°C, 24 h). Copper (8.5 ± 0.5 μg/L), zinc (15.2 ± 1.0 μg/L), and aluminum (24.8 ± 1.5 μg/L) all remained well below WHO drinking water standards at pH 8.0, suggesting adequate safety for wastewater treatment applications. Future validation is necessary due to several critical limitations, such as: (1) laboratory-scale validation only using synthetic solutions—real wastewater contains competing ions and organic matter that could reduce efficiency by 15–25%; (2) modest capacity limiting applicability to specific concentration ranges; (3) economic projections requiring pilot-scale validation (6–12 months); (4) scale-up uncertainties from batch to continuous fixed-bed operation; and (5) an unclear regulatory pathway for waste-derived materials in water treatment. Authentic wastewater validation, pilot-scale column investigations, process integration evaluation, regenerant phosphate recovery techniques, and comparative life cycle analysis are among the upcoming priorities. This study establishes laboratory-scale proof-of-concept for LTSSC-based phosphate adsorption as a promising sustainable technology addressing dual environmental challenges: hazardous waste disposal and eutrophication control, contributing to the principles of the circular economy and UN Sustainable Development Goals 6, 12, and 13, despite acknowledged limitations.

## Data Availability

The datasets generated and/or analyzed during the current study are available from the corresponding author on reasonable request.
